# Antioxidant and Anti-Aging Activity of *Helichrysum arenarium* (L.) Moench: Comparative Evaluation of Extracts from Shoots, Inflorescences, Plantlets and Callus Tissue

**DOI:** 10.3390/molecules31152625

**Published:** 2026-07-28

**Authors:** Joanna Jabłońska, Maciej Obrębski, Rafał M. Kiełkiewicz, Irena Macias, Julia Bogusz, Weronika Skowrońska, Katarzyna Sykłowska-Baranek

**Affiliations:** 1 Department of Pharmaceutical Biology, Faculty of Pharmacy, Medical University of Warsaw, 1 Banacha St., 02-097 Warsaw, Poland; s082470@student.wum.edu.pl (J.J.); maciej.obrebski@wum.edu.pl (M.O.); s091804@student.wum.edu.pl (I.M.); weronika.skowronska@wum.edu.pl (W.S.); katarzyna.syklowska-baranek@wum.edu.pl (K.S.-B.); 2 MicrobiotaLab, Department of Pharmaceutical Microbiology and Bioanalysis, Faculty of Pharmacy, Medical University of Warsaw, 1 Banacha St., 02-097 Warsaw, Poland; 3 Faculty of Biochemistry, Biophysics and Biotechnology at the Jagiellonian University, 7 Gronostajowa St., 30-387 Cracow, Poland; julia.bogusz@student.uj.edu.pl

**Keywords:** sandy everlasting, secondary metabolites, plant tissue culture, HPLC-MS/MS, phytochemical profiling, collagenase and hyaluronidase inhibition, skin cells, anti-inflammatory activity, wound-healing properties

## Abstract

*Helichrysum arenarium* (L.) Moench is a European native species typically associated with dry, sunny, sandy, and nutrient-poor habitats. In European herbal practice, preparations from *H. arenarium* inflorescences are traditionally used for the symptomatic relief of digestive complaints. Polyphenolic constituents, particularly flavonoid and chalcone derivatives, are among the most characteristic compounds detected in the inflorescences. Intensive harvesting and degradation of natural habitats have increased pressure on wild populations. Since *H. arenarium* is legally protected in many countries, biotechnological approaches for plant biomass production represent a promising alternative. In the present study, shoot and callus cultures were established, and detailed extract profiling was performed using ultra-high-performance liquid chromatography coupled with diode-array detection and electrospray ionization multistage mass spectrometry (UHPLC-DAD-ESI-MS^3^). Cytotoxic, antioxidant, anti-inflammatory, wound-healing, anti-collagenase, and anti-hyaluronidase activities were also evaluated. In vitro-derived materials were compared with inflorescences and shoots of the maternal plant. The analyzed materials showed pronounced tissue-dependent metabolic differentiation. The highest callus growth rate was observed for the Ha-C-1 line, which also accumulated the highest levels of chlorogenic acid (25.80 ± 3.09 mg/g dry extract) and isochlorogenic acid A (55.83 ± 13.78 mg/g dry extract). Apigenin was detected in the inflorescence extract, reaching 3.55 ± 1.42 mg/g dry extract. The extracts did not markedly affect HaCaT viability at 15.63–500 µg/mL, while the strongest bioactivities were observed for inflorescence and maternal plant extracts.

## 1. Introduction

*Helichrysum arenarium* (L.) Moench, commonly known as sandy everlasting, is a perennial herb belonging to the Asterales order, the Asteraceae family, and the genus *Helichrysum*, which consists of 564 species of flowering plants, native to Europe, Central Asia, and parts of China [1]. The species is typically associated with dry, sunny, sandy, and nutrient-poor habitats, and it is readily recognized by yellow capitula that retain their color after drying [2]. Many representatives of this genus have been incorporated into traditional medical practices and are currently being examined as sources of specialized metabolites with potential pharmaceutical, nutraceutical, and cosmeceutical relevance. In European herbal practice, preparations obtained from *H. arenarium* inflorescences have mainly been associated with the management of digestive and hepatobiliary complaints. Their traditional indications include support of bile secretion and bile flow, as well as reported mild spasmolytic, diuretic, detoxifying, and hepatoprotective uses [2,3]. The European Medicines Agency recognizes the traditional use of *H. arenarium* flower preparations for the symptomatic relief of digestive complaints, including sensations of fullness and bloating [4].

The biological activity of *H. arenarium* is linked to a chemically diverse spectrum of specialized metabolites. Polyphenolic constituents, particularly flavonoid and chalcone derivatives, are among the most characteristic compounds detected in the inflorescences. These metabolites occur both as free aglycones and as glycosides and include representatives of flavanones, flavones, flavonols, and chalcones. Isosalipurposide, a chalcone glycoside responsible in part for the yellow color of the flower heads, is widely regarded as one of the principal marker compounds of the species. Naringenin and its glycosides, salipurposide, quercetin, quercitrin, isoquercitrin, kaempferol, apigenin, luteolin, and related derivatives have also been reported [2,5,6]. Phenolic acids constitute another important fraction of the *H. arenarium* metabolome. Chlorogenic acid, caffeic acid derivatives, dicaffeoylquinic acids, and derivatives of ferulic and *p*-coumaric acids have been identified in flowers and aerial parts [2,6]. In addition to these dominant phenolic groups, other classes of constituents, including coumarins, phthalide- and pyrone-type compounds, proanthocyanidins, sterols, lignans, triterpenoids, carotenoids, fatty acids, and aromatic glycosides, have been described. The volatile fraction is usually present at relatively low levels and may contain mono- and sesquiterpenes, although its qualitative and quantitative composition is strongly influenced by geographical origin, developmental stage, environmental conditions, and extraction procedure [2]. The inflorescences of *H. arenarium* have repeatedly been characterized as polyphenol-rich plant material with antioxidant potential. Methanolic and hydroethanolic extracts have demonstrated radical-scavenging and reducing activities, and these effects have commonly been associated with their total phenolic and flavonoid contents [6,7]. Isolated flavanone and chalcone glycosides, including arenariumosides, have also been evaluated for their ability to modulate inflammatory processes, including tumor necrosis factor-α-related responses [5]. Other experimentally reported properties of *H. arenarium* extracts include anti-inflammatory, antimicrobial, spasmolytic, hepatoprotective, and choleretic effects, although the magnitude of these activities depends on the plant organ, extract composition, solvent, and experimental model [2,5,6,7].

The pharmacological potential of the genus is not restricted to *H. arenarium*. *Helichrysum italicum* is one of the best-investigated species and has traditionally been used in Mediterranean medicine for inflammatory conditions, infections, wounds, bruises, and skin disorders. Its extracts, essential oils, and hydrolates have demonstrated antioxidant, antimicrobial, anti-inflammatory, and regenerative effects [6,8]. In experimental wound-healing models, *H. italicum*-derived preparations have been reported to promote collagen deposition and support regenerative responses of fibroblasts and skin stem cell-based models [9]. In addition, prenylated chalcones and flavonoids isolated from *Helichrysum teretifolium* have demonstrated antioxidant activity and inhibition effects on selected enzymes associated with skin aging, supporting the broader potential of the genus as a source of skin-protective compounds [10].

Oxidative stress is a key factor in skin aging, promoting cellular damage and extracellular matrix degradation. Collagenase and hyaluronidase contribute to the breakdown of collagen and hyaluronic acid, leading to reduced skin firmness, hydration, and elasticity. Therefore, plant-derived compounds with antioxidant and enzyme-inhibitory properties are of considerable interest for skin-protective and anti-aging applications.

The occurrence of phenolic acids, flavonoids, and chalcones in *Helichrysum* species provides a rationale for assessing these activities in *H. arenarium* material obtained from different sources [9,10]. Despite its medicinal value, the sustainable acquisition of *H. arenarium* biomass remains challenging. Intensive collection of the inflorescences and degradation of natural habitats have contributed to pressure on wild populations. In Poland, *H. arenarium* is subject to partial legal protection, and the acquisition of material from natural populations is legally restricted [11]. Therefore, the use of wild-growing plants for scientific or industrial purposes may require appropriate authorization [12], as was the case for the material used in the present study. The conservation status of the species highlights the need to develop reproducible alternative sources of biomass that would reduce dependence on material collected from natural habitats.

Plant tissue culture offers a sustainable alternative for biomass production under controlled conditions, independent of environmental and seasonal limitations. In vitro systems allow the selection of productive lines and the enhancement of specialized metabolite biosynthesis through various biotechnological approaches. Callus cultures are particularly valuable as rapidly proliferating, renewable tissues suitable for metabolite production and further culture development.

Previous studies have demonstrated the feasibility of establishing in vitro cultures within the genus *Helichrysum*. Shoot regeneration and callus formation have been reported for *H. italicum* and *Helichrysum stoechas*, and the resulting cell masses have been proposed as material for suspension cultures and studies on specialized metabolites [13]. Micropropagation protocols have also been developed for *H. arenarium*, showing that the species can be effectively multiplied, rooted, and acclimatized under controlled conditions [12]. In vitro-derived seedlings have subsequently been cultivated under field conditions, confirming that biotechnological propagation may support both conservation and agricultural production of medicinal raw material [14]. Nevertheless, the available studies concerning *H. arenarium* have focused primarily on micropropagation, acclimatization, and field cultivation. Information on the phytochemical composition and biological properties of its callus cultures remains limited, particularly when callus tissues are directly compared with inflorescences, aerial parts of mother plants, and differentiated plantlets maintained in vitro.

Based on available evidence, it was hypothesized that *H. arenarium* callus cultures, despite their undifferentiated nature, would retain the ability to produce phenolic compounds and exhibit antioxidant, collagenase- and hyaluronidase-inhibitory, wound-healing, and anti-inflammatory activities. These properties were expected to vary among in vitro lines and according to the degree of tissue differentiation. Therefore, the aim of this study was to establish shoot and callus cultures of *H. arenarium* and compare their phytochemical composition and biological activities with those of inflorescences and aerial parts of mother plants. Methanolic extracts from mother plants, three in vitro shoot lines, and their corresponding callus cultures were analyzed by UHPLC-DAD-ESI-MS^3^ and evaluated for phenolic and flavonoid contents, antioxidant capacity, collagenase- and hyaluronidase-inhibitory activities, wound-healing potential, effects on immortalized human keratinocytes, and anti-inflammatory activity based on IL-6 and IL-8 levels.

## 2. Results and Discussion

### 2.1. Establishment and Growth of in Vitro Plantlet and Callus Cultures

**Figure 1:**
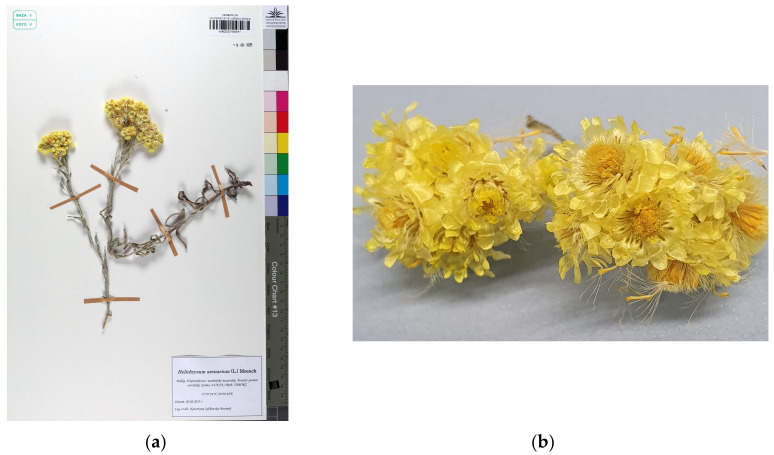
*H. arenarium* plant collected from natural habitat (**a**) and its inflorescences (**b**).

**Figure 2:**
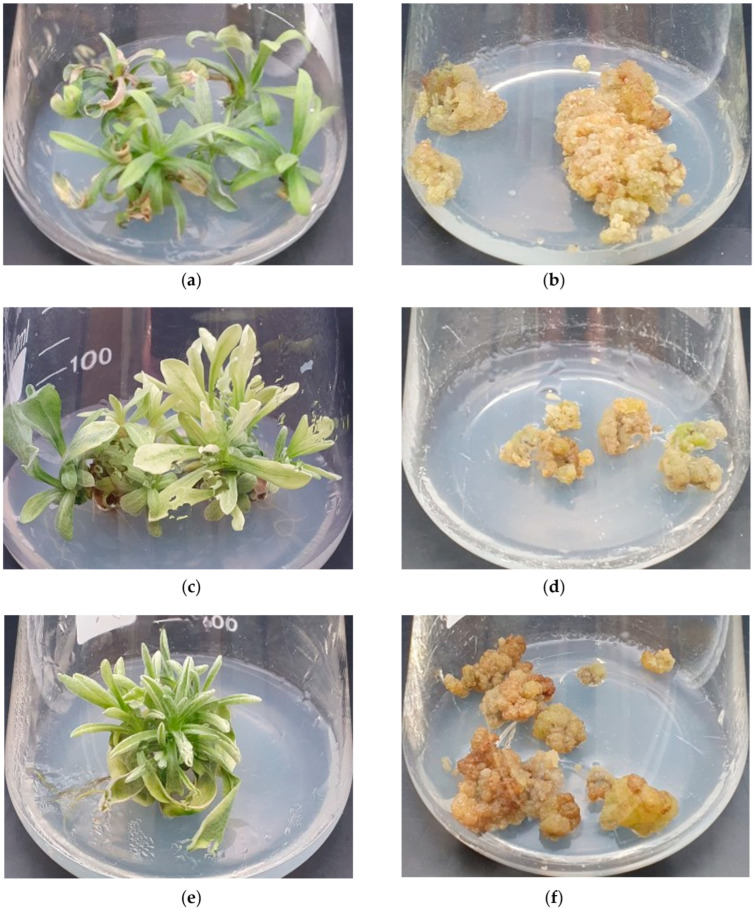
*H. arenarium* plantlets and corresponding callus cultures: (**a**) plantlet line Ha-P-1; (**b**) callus line Ha-C-1; (**c**) plantlet line Ha-P-4; (**d**) callus line Ha-C-4; (**e**) plantlet line Ha-P-12; and (**f**) callus line Ha-C-12. Plantlets were cultivated on hormone-free Murashige and Skoog (MS) medium, whereas callus cultures were maintained for 4 weeks on MS medium supplemented with 1 mg/L 6-benzylaminopurine (BAP) and 1 mg/L 2,4-dichlorophenoxyacetic acid (2,4-D).

*H. arenarium* in vitro cultures were established using 20 seeds from plants collected from the natural habitat (Figure 1). After four weeks of culture on a half-strength hormone-free Murashige and Skoog medium [15], 16 seeds produced seedlings that gave origin to 16 shoot lines. Three of them, i.e., Ha-P-1, Ha-P-4 and Ha-P-12, were chosen for further investigations (Figure 2a, Figure 2c and Figure 2e, respectively). Callus cultures were initiated starting from leaves that were cut off and placed on solid MS medium supplemented with benzylaminopurine (BAP) 1 mg/L and 2,4-dichlorophenoxyacetic acid (2,4-D) 1 mg/L. Within four weeks of culture, callus developed on the leaf surface, which was used in subsequent passages to develop callus cultures of three lines, i.e., Ha-C-1, Ha-C-4 and Ha-C-12 (Figure 2b, Figure 2d and Figure 2f, respectively).

**Figure 3:**
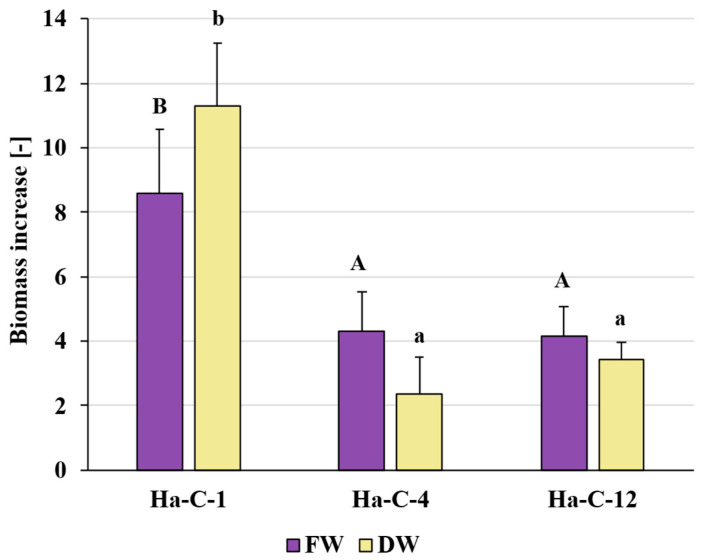
*H. arenarium* callus growth rate of fresh weight (FW) and dry weight (DW). Data are presented as mean ± SD (*n* = 3). Different letters above the bars indicate statistically significant differences between groups according to one-way ANOVA followed by Tukey’s HSD post hoc test (*p* < 0.05). Ha-C-1, Ha-C-4, and Ha-C-12 denote *H. arenarium* callus lines 1, 4, and 12, respectively. The reports concerning callus culture of *H. arenarium* are very limited; the majority of the are focused on micropropagation of this species, which is classified in many countries as a species in danger due to overexploitation of its natural habitats [3,11,14,16]. To date, there is one study reporting induction of callus on MS medium with addition of 2,4-D 1 mg/L [16]; however, the proliferation of the resulting cell biomass was not investigated.

The callus biomass growth was determined and expressed as a ratio of final weight to initial weight (Figure 3). The highest increases in both fresh and dry biomass were observed for the Ha-C-1 callus line and amounted to 8.5 ± 2.0-fold and 11.3 ± 2.0-fold, respectively.

The current study aimed to develop and examine callus and plantlet cultures in relation to their phytochemical composition and biological properties. The cultivation of plantlets was carried out; however, they served as a source of callus tissue that, from the biotechnological point of view, could be further scaled up via suspension cultures as a platform for commercial-scale production of biologically active compounds.

### 2.2. Phytochemical Characterization of H. arenarium Extracts

#### 2.2.1. Qualitative UHPLC-DAD-ESI-MS^3^ Profiling of Specialized Metabolites

**Figure 4:**
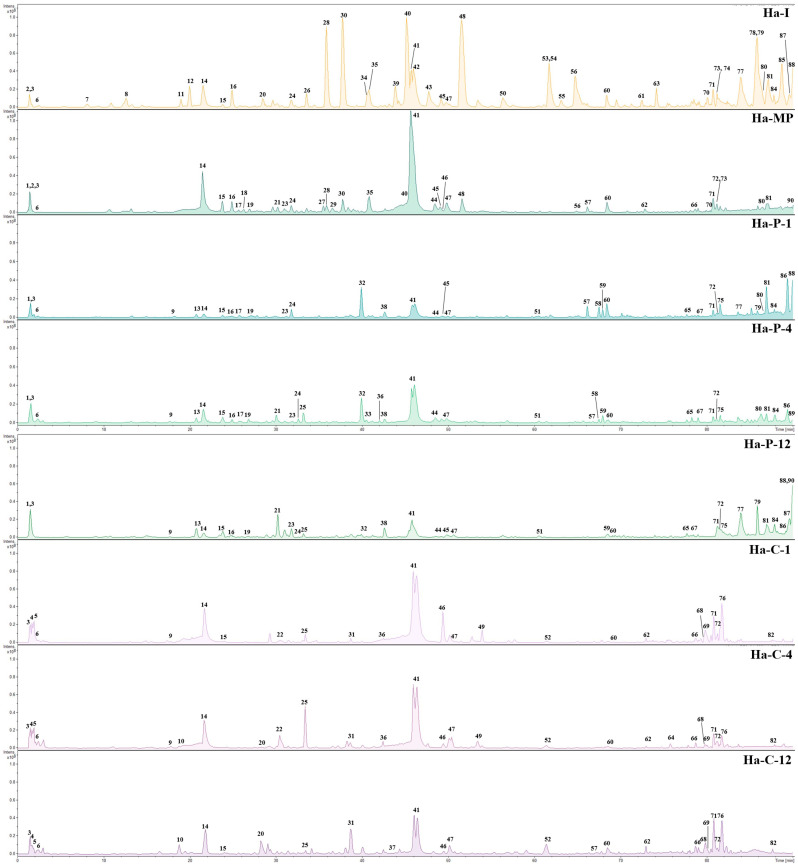
Base peak chromatograms (BPCs) recorded in negative ion mode for methanolic extracts of *H. arenarium*: inflorescences (Ha-I), maternal plants (Ha-MP), plantlet lines (Ha-P-1; Ha-P-4; Ha-P-12) and callus tissue lines (Ha-C-1; Ha-C-4; Ha-C-12). Peak numbers correspond to compounds listed in Table 1.

In the current study, the detailed analysis using UHPLC-DAD-ESI-MS^3^ of methanolic extracts of callus (Ha-C-1, Ha-C-4, Ha-C-12), plantlets (Ha-P-1, Ha-P-4, Ha-P-12), maternal plant (Ha-MP) shoots, and inflorescence (Ha-I) was performed (Figure 4). The phytochemical profiling revealed a chemically diverse metabolite pattern in the analyzed *H. arenarium* extracts, including saccharides, phenolic acids and quinic acid derivatives, chalcones, flavanones, flavones, flavonols, flavanonols, α-pyrones, phthalides, spiroketal-type compounds, and other specialized metabolites. Chlorogenic acid, isochlorogenic acid A, and apigenin were confirmed using authentic reference standards. All other named compounds listed in Table 1 were tentatively identified based on their UV–Vis and MS^2^/MS^3^ characteristics, chromatographic behavior, and literature data, whereas signals without a reliable structural assignment were retained as unknowns. Representative MS^2^/MS^3^ spectra supporting the tentative identifications are provided in Figures S1–S12. The identified and tentatively assigned compounds were unevenly distributed among analyzed types of plant materials, indicating pronounced tissue-dependent metabolic differentiation. Compounds that could not be reliably assigned were retained as unknowns in Table 1 and were not included in the class-specific discussion.

**Figure 5:**
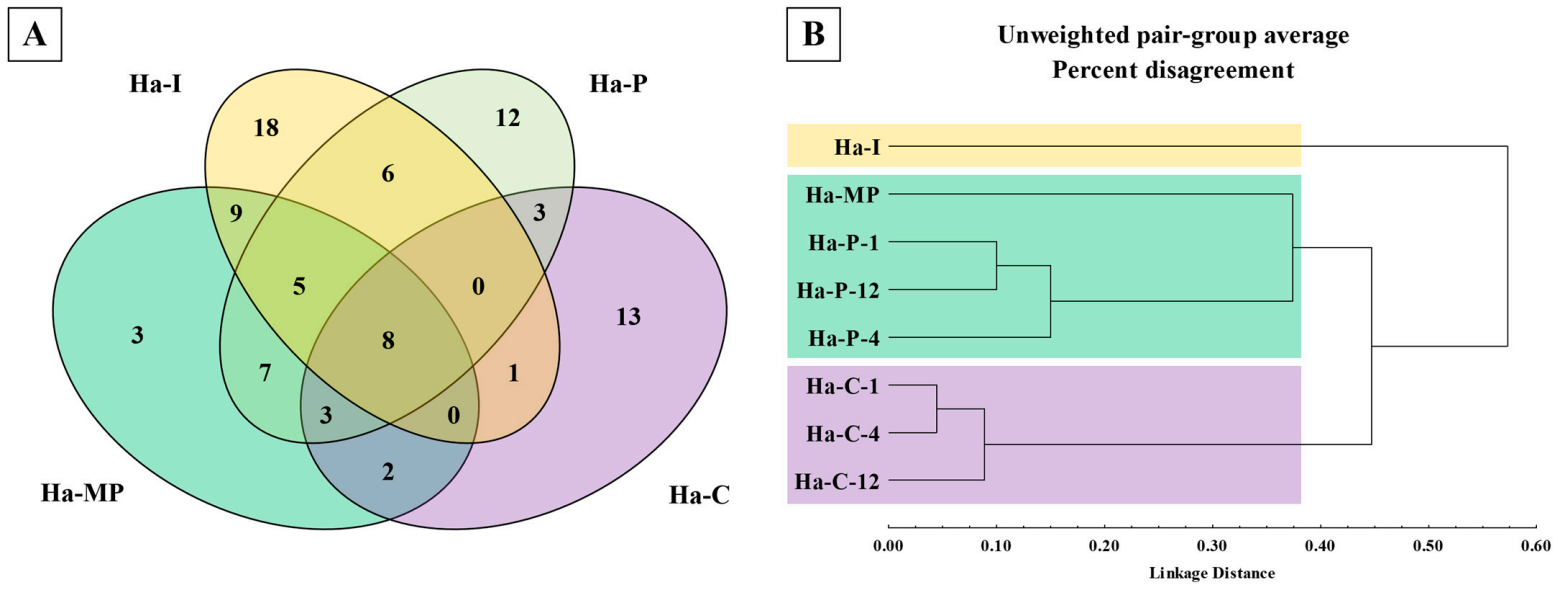
Comparative evaluation of phytochemical profile diversity in *H. arenarium* inflorescences (Ha-I), aerial parts of mother plants (Ha-MP), in vitro-derived plantlets (Ha-P), and callus lines (Ha-C). (**A**) Venn diagram based on metabolite presence/absence data, showing the distribution of detected metabolites among the analyzed plant materials, with plantlet and callus samples considered within their respective groups. (**B**) Dendrogram showing hierarchical clustering of individual samples based on binary metabolite presence/absence profiles. Clustering was performed using the unweighted pair-group method with arithmetic mean (UPGMA) and percent disagreement as the distance metric. Unidentified compounds were included in the analysis.

Based on the presence/absence of all detected metabolites in all analyzed plant material types, a comparative statistical analysis was performed, and a Venn diagram together with a hierarchical clustering dendrogram were prepared. For the Venn diagram (Figure 5A), all data for metabolites detected in the in vitro-derived plantlet samples were grouped as Ha-P, and all for callus samples were grouped as Ha-C. Unidentified compounds were also included in the analysis as separate entries. Eight metabolites were found common to all analyzed material types [e.g., chlorogenic acid, isochlorogenic acids B and C, or 6-hydroxy-7-methoxy-2-(2-phenylethyl)chromone], indicating the presence of a shared metabolic core in *H. arenarium*. At the same time, clear differences were observed among the investigated materials. The highest number of unique metabolites was found in the inflorescence extract Ha-I, with 18 metabolites detected exclusively in this group. Callus cultures also showed a distinct profile, with 13 metabolites present only in Ha-C, whereas 12 metabolites were specific to Ha-P and only three to Ha-MP.

Hierarchical clustering (Figure 5B) based on the same presence/absence dataset separated the samples according to the type of plant material. Callus cultures formed a distinct cluster, with Ha-C-1 and Ha-C-4 showing the highest similarity, followed by Ha-C-12. In vitro-derived plantlets also clustered together, with Ha-P-1 and Ha-P-12 being the most similar, while Ha-P-4 joined this subgroup at a slightly greater linkage distance. The ex vitro mother plant material Ha-MP, was closer to the plantlet cluster than to callus cultures, whereas Ha-I showed the most distinct metabolite profile and separated from the remaining samples at the highest linkage distance.

**Figure 6:**
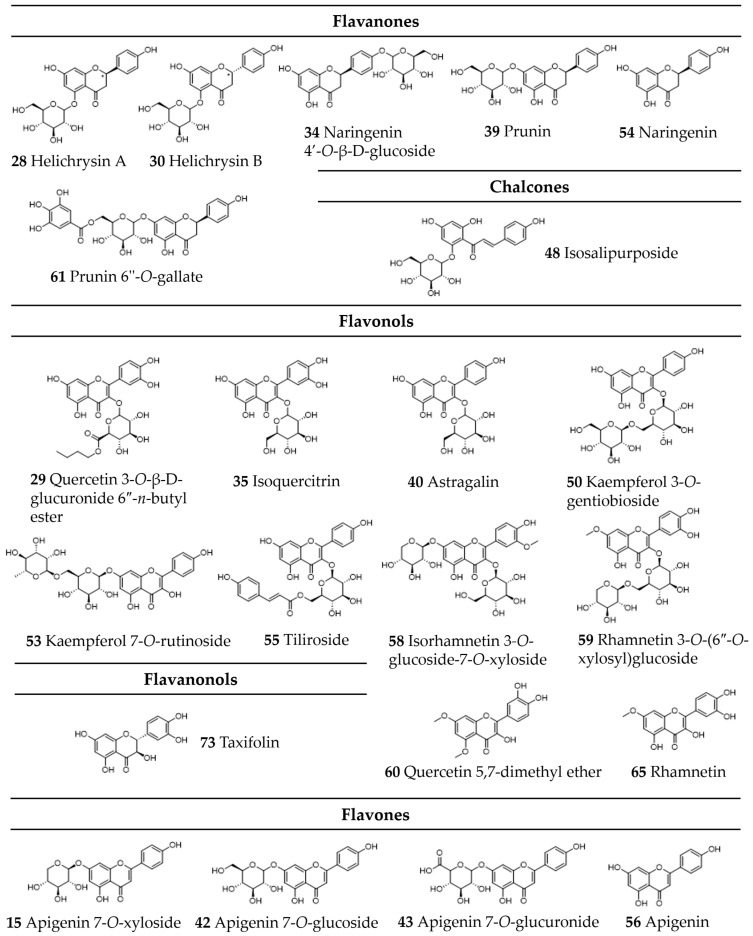

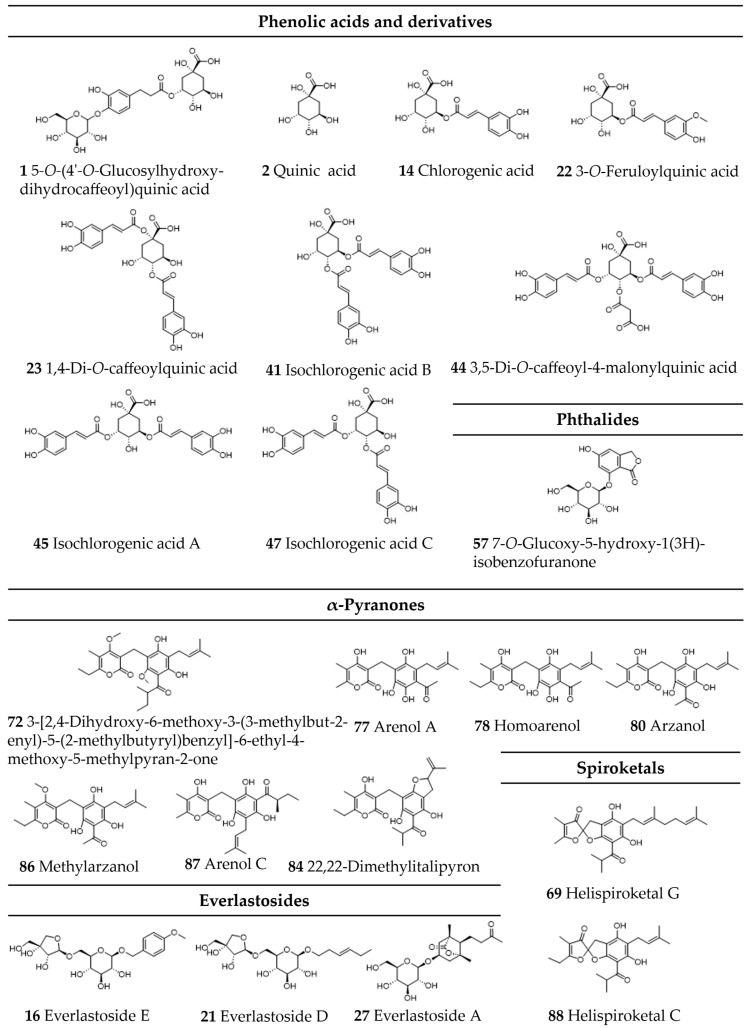

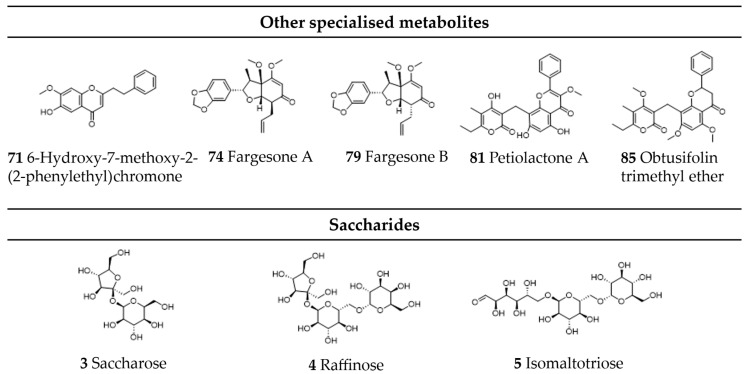
Chemical structures of all confirmed or tentatively identified metabolites in *H. arenarium* callus, plantlets, maternal plant shoots and inflorescence.

Overall, these results indicate that the phytochemical profile of *H. arenarium* strongly depended on the type of plant material. In vitro-derived plantlets partially retained the metabolite composition of the ex vitro mother plant, whereas callus cultures developed a more distinct chemical profile. To complement the phytochemical profiling results, the chemical structures of the compounds identified or tentatively identified in the analyzed extracts and assigned to appropriate metabolite groups are presented below in Figure 6.

##### Chalcones

Chalcones are open-chain flavonoid precursors and an important subgroup of phenolic compounds in Asteraceae, often associated with antioxidant, anti-inflammatory, and hepatoprotective properties [35]. In *H. arenarium*, isosalipurposide is regarded as a characteristic chalcone marker of the inflorescences and has been reported as one of the dominant compounds of the species [2,3].

In the present study, only isosalipurposide (chalconaringenin-2′-*O*-glucoside, **C_48_**) was identified from the group. It was detected at t_R_ 51.6 min and showed a deprotonated molecular ion at *m*/*z* 433, with a major fragment ion at *m*/*z* 271, corresponding to the loss of a hexose moiety and formation of the chalconaringenin aglycone. Negative-ion MS^2^ spectrum of compound **C_48_**, with the proposed structures of the major product ions, is shown in Figure S1. This compound was present only in the ex vitro samples, namely in the inflorescence extract Ha-I and in the mother plant extract Ha-MP. Notably, isosalipurposide accounted for one of the most prominent peaks in the chromatogram of the inflorescence extract, indicating its importance as a major specialized metabolite in the phytochemical profile of *H. arenarium* flowers.

Its absence from in vitro-derived plantlets and callus cultures suggests that the biosynthesis of this compound may be strongly dependent on tissue differentiation, developmental stage, or environmental conditions, and may not be efficiently expressed under the applied in vitro culture conditions. Thus, the tested in vitro systems were not able to reproduce this particular feature of the ex vitro phytochemical profile. Nevertheless, the cultures retained the capacity to synthesize other important groups of specialized metabolites characteristic of the species, as described in the following sections.

##### Flavanones

Flavanones are a subclass of flavonoids characterized by a C6-C3-C6 carbon skeleton, a closed heterocyclic C ring, a carbonyl group at C-4, and a saturated C2-C3 bond, which distinguishes them from flavones and flavonols [36,37]. They are biosynthetically derived from chalcones and may serve as intermediates in the formation of other flavonoid subclasses [36]. Flavanones are widely distributed in plants and are commonly associated with antioxidant, anti-inflammatory, and cytoprotective activities, particularly in the case of naringenin and its glycosides [38]. In *H. arenarium*, naringenin and its glycosides are among the characteristic and repeatedly reported flavanone-type constituents of the species [2,3].

In the present study, seven flavanone-type compounds were identified or tentatively assigned. These included helichrysin A/(+)-naringenin 5-*O*-β-D-glucoside (**C_28_**), helichrysin B/salipurposide/(−)-naringenin 5-*O*-β-D-glucoside (**C_30_**), naringenin 4′-*O*-β-D-glucoside (**C_34_**), prunin/naringenin 7-*O*-β-D-glucoside (**C_39_**), free naringenin (**C_54_**), prunin 6″-*O*-gallate/naringenin 7-*O*-β-D-(6″-*O*-galloyl)glucoside (**C_61_**), and partially identified prunin derivative (**C_63_**). The assignment of **C_28_** and **C_30_** as two naringenin 5-*O*-glucoside diastereoisomers is consistent with previous phytochemical reports on *H. arenarium* [2]. The assigned flavanones showed characteristic fragmentation patterns, mainly involving the loss of glycosidic residues and formation of the naringenin aglycone ion at *m*/*z* 271, followed by diagnostic product ions such as *m*/*z* 177, 151, and 107. Additionally, to support the tentative assignment of the structurally more complex galloylated derivative **C_61_**, its negative-ion MS^2^ and MS^3^ spectra, together with the proposed fragmentation pathway and structures of selected product ions, were provided in Figure S2.

Flavanones were detected exclusively in ex vitro-derived material. Helichrysin A and helichrysin B were present in both the inflorescence extract Ha-I and the mother plant extract Ha-MP. In contrast, free naringenin and its glycosides: naringenin 4′-*O*-glucoside, prunin, prunin 6″-*O*-gallate, and the prunin derivative were detected only in Ha-I. None of the assigned flavanones was observed in the analyzed in vitro-derived plantlets or callus cultures.

The restriction of naringenin-derived flavanones to Ha-I and Ha-MP confirms that this fraction is mainly associated with differentiated ex vitro material. The particularly diverse flavanone profile of Ha-I supports the typical chemical character of the traditionally used flower material, whereas the absence of these compounds in plantlets and callus cultures indicates a marked reduction in this branch of flavonoid metabolism under the tested in vitro conditions.

##### Flavones

Flavones are a subclass of flavonoids characterized by a C6-C3-C6 carbon skeleton, a carbonyl group at C-4, and a double bond between C-2 and C-3 in the heterocyclic C ring. In contrast to flavonols, flavones typically lack a hydroxyl group at C-3, while the presence and position of hydroxyl, methoxy, and glycosidic substituents determine their chromatographic and spectrometric properties. They are biosynthetically formed from flavanones and are widely distributed in plants as free aglycones and glycosides [3]. Flavones, especially apigenin and luteolin derivatives, are commonly associated with antioxidant, anti-inflammatory, cytoprotective, and enzyme-modulating properties [36,37]. In *H. arenarium*, apigenin and apigenin glycosides have been previously reported as part of the characteristic flavonoid fraction of the species [2,3].

In the present study, four flavone-type compounds were identified or tentatively assigned: apigenin 7-*O*-xyloside (**C_15_**), apigenin 7-*O*-glucoside (**C_42_**), apigenin 7-*O*-glucuronide (**C_43_**), and apigenin (**C_56_**). The glycosidic apigenin derivatives showed deprotonated molecular ions followed by the formation of the apigenin aglycone ion at *m*/*z* 269, corresponding to the loss of sugar or uronic acid residues. Apigenin 7-*O*-xyloside showed [M−H]^−^ at *m*/*z* 401 and a fragment ion at *m*/*z* 269 formed by neutral loss of 132 Da, consistent with the loss of a pentose moiety. Apigenin 7-*O*-glucoside showed [M−H]^−^ at *m*/*z* 431 and a major product ion at *m*/*z* 269, and neutral loss of 162 Da corresponding to the loss of a hexose residue. Apigenin 7-*O*-glucuronide showed [M−H]^−^ at *m*/*z* 445 and fragment ions at *m*/*z* 269 and 175, consistent with the loss of a glucuronic acid residue (Figure S3). Free apigenin was detected at *m*/*z* 269 and produced characteristic fragment ions at *m*/*z* 225 and 159.

The distribution of flavones differed among the analyzed extracts. Apigenin 7-*O*-xyloside (**C_15_**) was the most widely distributed and was detected in Ha-I, Ha-MP, all three in vitro-derived plantlet extracts, and two callus lines, Ha-C-1 and Ha-C-12. In contrast, apigenin 7-*O*-glucoside (**C_42_**) and apigenin 7-*O*-glucuronide (**C_43_**) were detected only in the inflorescence extract. Free apigenin (**C_56_**) was present in the ex vitro-derived extracts Ha-I and Ha-MP, but was not detected in plantlet or callus extracts.

The contrasting distribution of apigenin 7-*O*-xyloside and the remaining apigenin derivatives suggests that individual flavone glycosides respond differently to tissue differentiation and culture conditions. While **C_15_** was retained in several in vitro-derived samples, apigenin 7-*O*-glucoside, apigenin 7-*O*-glucuronide, and free apigenin were restricted to ex vitro material. This indicates that selected flavone pathways remain active in cultured biomass, but the complete apigenin profile of the inflorescences is not simply reproduced in the tested culture conditions.

##### Flavonols

Flavonols are a subclass of flavonoids characterized by the C6-C3-C6 carbon skeleton, a double bond between C-2 and C-3, a carbonyl group at C-4, and a hydroxyl group at C-3 of the heterocyclic C ring. This 3-hydroxyflavone backbone distinguishes flavonols from flavones and strongly influences their antioxidant potential, metal-chelating capacity, UV-absorbing properties, and biological activity [36,37]. Flavonols commonly occur in plants as O-glycosides, acylated glycosides, methylated derivatives, and free aglycones. In *H. arenarium*, flavonols have been reported as part of the characteristic phenolic fraction, mainly as kaempferol and quercetin derivatives, including astragalin and isoquercitrin [2,3].

In the analyzed plant materials ten flavonol-type compounds were identified or tentatively assigned: quercetin 3-*O*-β-D-glucuronide 6″-n-butyl ester (**C_29_**), isoquercitrin/quercetin 3-*O*-β-D-glucoside (**C_35_**), astragalin/kaempferol 3-*O*-glucopyranoside (**C_40_**), kaempferol 3-*O*-gentiobioside (**C_50_**), kaempferol 7-*O*-rutinoside (**C_53_**), tiliroside/kaempferol 3-*O*-(6″-*O*-*p*-coumaroyl)-β-D-glucoside (**C_55_**), isorhamnetin 3-*O*-glucoside-7-*O*-xyloside (**C_58_**), rhamnetin 3-*O*-(6″-*O*-xylosyl)glucoside (**C_59_**), quercetin 5,7-dimethyl ether (**C_60_**), and rhamnetin (**C_65_**).

Quercetin-derived flavonols were represented by **C_29_**, **C_35_**, and **C_60_**. Quercetin 3-*O*-β-D-glucuronide 6″-n-butyl ester was detected at tR 36.7 min and showed [M−H]^−^ at *m*/*z* 533, with fragment ions at *m*/*z* 463 and 301. Isoquercitrin showed [M−H]^−^ at *m*/*z* 463 and a major fragment ion at *m*/*z* 301, corresponding to the loss of a hexose moiety (Figure S4). The later-eluting compound showing [M−H]^−^ at *m*/*z* 329, with fragment ions at *m*/*z* 311, 293, 229, 211, was assigned as quercetin 5,7-dimethyl ether.

Kaempferol-derived compounds were represented by **C_40_**, **C_50_**, **C_53_**, and **C_55_**. Astragalin showed [M−H]^−^ at *m*/*z* 447, with fragment ions at *m*/*z* 285, 327, and 255. Kaempferol 3-*O*-gentiobioside showed [M−H]^−^ at *m*/*z* 609 and fragment ions at *m*/*z* 447, 323, and 285, indicating cleavage of a disaccharide moiety and formation of the kaempferol aglycone ion. Kaempferol 7-*O*-rutinoside and tiliroside both showed [M−H]^−^ at *m*/*z* 593 and fragment ions at *m*/*z* 447 and 285, but differed in their chromatographic behavior and UV–Vis characteristics, supporting their assignment as distinct kaempferol derivatives (Figure S5).

Methylated flavonol derivatives were represented by **C_58_**, **C_59_**, **C_60_**, and **C_65_**. Isorhamnetin 3-*O*-glucoside-7-*O*-xyloside was detected at t_R_ 67.5 min and showed [M−H]^−^ at *m*/*z* 609, with fragment ions at *m*/*z* 477, 447, and 315, consistent with the independent cleavages of hexose and xylose moieties and formation of an isorhamnetin-type aglycone ion. Rhamnetin 3-*O*-(6″-*O*-xylosyl)glucoside also showed [M−H]^−^ at *m*/*z* 609, with a major fragment ion at *m*/*z* 315. Free rhamnetin was also detected later at t_R_ 77.7 min, showed [M−H]^−^ at *m*/*z* 315, and produced fragment ions at *m*/*z* 297, 279, and 171.

The distribution of flavonols differed markedly among the investigated extracts. Some compounds were associated mainly with ex vitro-derived material. Isoquercitrin and astragalin were detected in both Ha-I and Ha-MP, whereas kaempferol 3-*O*-gentiobioside, kaempferol 7-*O*-rutinoside, and tiliroside were restricted to the inflorescence extract Ha-I. Quercetin 3-*O*-β-D-glucuronide 6″-n-butyl ester was detected only in the mother plant extract. In contrast, isorhamnetin 3-*O*-glucoside-7-*O*-xyloside, rhamnetin 3-*O*-(6″-*O*-xylosyl)glucoside, and rhamnetin were characteristic of in vitro-derived plantlets. **C_58_** was detected in Ha-P-1 and Ha-P-4, whereas **C_59_** and **C_65_** were present in all three plantlet lines. Quercetin 5,7-dimethyl ether was the only flavonol-type compound detected in all analyzed extracts.

Flavonols showed a clearly tissue-dependent pattern. The inflorescence extract was enriched in kaempferol glycosides, including tiliroside and kaempferol 7-*O*-rutinoside, whereas the mother plant extract shared selected quercetin- and kaempferol-type compounds with the inflorescences and additionally contained **C_29_**. In vitro-derived plantlets were distinguished by methylated flavonols, particularly rhamnetin- and isorhamnetin-type derivatives. By contrast, callus cultures displayed a simplified flavonol profile limited to quercetin 5,7-dimethyl ether, suggesting that flavonol biosynthesis was retained under in vitro conditions but substantially redirected depending on tissue type.

##### Flavanonols

Flavanonols, also referred to as dihydroflavonols, are a subclass of flavonoids structurally related to flavonols, but lacking the C2=C3 double bond in the heterocyclic C ring. This structural feature makes them intermediate-type flavonoids between flavanones and flavonols. Taxifolin, also known as dihydroquercetin, is one of the best-known representatives of this group in the *Helichrysum* genus and has been associated with antioxidant, anti-inflammatory, and cytoprotective properties [36,37].

In the present study, one flavanonol-type compound was identified, namely taxifolin/dihydroquercetin (**C_73_**). It was detected at t_R_ 81.2 min and showed a deprotonated molecular ion at *m*/*z* 303, with product ions at *m*/*z* 259 and 179, supporting its assignment as a dihydroquercetin-type compound. Taxifolin was detected only in ex vitro-derived materials—inflorescence extract Ha-I and in the mother plant extract Ha-MP. This distribution supports the association of dihydroquercetin-type metabolism with differentiated plant tissues and indicates that this minor flavonoid branch was not maintained in the analyzed plantlet and callus cultures.

##### Phenolic Acids and Derivatives

Phenolic acids are an important class of plant-specialized metabolites, commonly represented by hydroxybenzoic and hydroxycinnamic acid derivatives. In Asteraceae and other phenolic-rich plant materials, hydroxycinnamic acids often occur as esters with quinic acid, including caffeoylquinic, feruloylquinic, and dicaffeoylquinic acids. These compounds are frequently associated with antioxidant, anti-inflammatory, and cytoprotective properties, mainly due to their phenolic hydroxyl groups and redox-active structure [39,40]. In *H. arenarium*, phenolic acids have been repeatedly reported as an important fraction of the metabolome, with chlorogenic acid described as the main hydroxycinnamic acid derivative and with caffeic, ferulic, and related acids also reported in the species [2,3].

In the UHPLC-DAD-ESI-MS^3^ analysis, nine phenolic-acid type compounds were identified or tentatively assigned: 5-*O*-(4′-*O*-glucosylhydroxydihydrocaffeoyl)quinic acid (**C_1_**), quinic acid (**C_2_**), chlorogenic acid/3-*O*-caffeoylquinic acid (**C_14_**), 3-*O*-feruloylquinic acid (**C_22_**), 1,4-di-*O*-caffeoylquinic acid (**C_23_**), 3,4-di-*O*-caffeoylquinic acid/isochlorogenic acid B (**C_41_**), 3,5-di-*O*-caffeoyl-4-malonylquinic acid (**C_44_**), 3,5-di-*O*-caffeoylquinic acid/isochlorogenic acid A (**C_45_**), and 4,5-di-*O*-caffeoylquinic acid/isochlorogenic acid C (**C_47_**). Although quinic acid itself is not a phenolic acid, it was discussed in this group because it represents the core structural unit of the caffeoylquinic, feruloylquinic, and dicaffeoylquinic acid derivatives detected in the analyzed extracts.

Chlorogenic acid showed a deprotonated molecular ion at *m*/*z* 353 and characteristic fragment ions at *m*/*z* 191 and 179, consistent with a caffeoylquinic acid structure. 3-*O*-Feruloylquinic acid showed [M−H]^−^ at *m*/*z* 367 and fragment ions at *m*/*z* 193, 191, and 173. The dicaffeoylquinic acid compounds: **C_23_**, **C_41_**, **C_45_**, and **C_47_** showed [M−H]^−^ at *m*/*z* 515 and produced diagnostic product ions at *m*/*z* 353, 191, 179, and 173, consistent with the cleavage of caffeoyl residues and formation of quinic-acid-related fragments. The malonylated derivative **C_44_** showed [M−H]^−^ at *m*/*z* 601, with fragment ions including *m*/*z* 557, 515, 439, 395, 233, 191, and 179, supporting its assignment as 3,5-di-*O*-caffeoyl-4-malonylquinic acid. Negative-ion MS^2^ and MS^3^ spectra of compound **C_44_**, together with the proposed fragmentation pathways and structures of the selected most abundant product ions, are shown in Figure S6.

Phenolic-acid-related compounds were widely represented in the analyzed *H. arenarium* extracts, but their distribution depended strongly on the type of biomass. Chlorogenic acid, isochlorogenic acid B and C were among the most broadly distributed compounds and were detected across ex vitro material, in vitro-derived plantlets, and callus cultures. Other derivatives, including 3-*O*-feruloylquinic acid, 1,4-di-*O*-caffeoylquinic acid, and 3,5-di-*O*-caffeoyl-4-malonylquinic acid, showed a more selective distribution among the investigated extracts. Isochlorogenic acid A was also an important member of this group and was sufficiently abundant for quantitative evaluation together with chlorogenic acid.

This profile indicates that phenolic acid metabolism is strongly retained in *H. arenarium* in vitro cultures. In contrast to chalcones and flavanones, which were mainly associated with ex vitro inflorescences, caffeoylquinic and dicaffeoylquinic acid derivatives were present not only in differentiated plant organs but also in plantlet and callus extracts. This suggests that the biosynthetic capacity for quinic-acid-derived hydroxycinnamates is maintained under in vitro conditions. The particularly high quantitative levels of chlorogenic acid and isochlorogenic acid A observed in selected callus lines indicate that undifferentiated *H. arenarium* biomass may redirect phenolic metabolism toward caffeoylquinic acid accumulation. Therefore, callus cultures should not be interpreted as chemically poor material, but rather as metabolically distinct biomass with potential value as a source of selected phenolic acids.

##### α-Pyranones

α-Pyranones, also referred to as α-pyrones or 2-pyrones, are lactones based on a pyran-2-one scaffold. This structural motif occurs in numerous natural products and is frequently associated with chemically diverse polyketide-derived metabolites. Selected α-pyrone derivatives have been reported to display biological activities, including antioxidant, antimicrobial, anti-inflammatory, cytotoxic, and enzyme-modulating effects, although these properties are compound-specific and should not be generalized to the whole class [41]. In *H. arenarium*, α-pyrone derivatives have been described among the yellow pigments of the inflorescences, including arenol and homoarenol, which supports the relevance of this class in the phytochemical profile of the species [2].

In the present study, seven α-pyranone-type compounds were tentatively identified based on the available literature data and observed MS/MS fragmentation characteristics: 3-[2,4-dihydroxy-6-methoxy-3-(3-methylbut-2-enyl)-5-(2-methylbutyryl)benzyl]-6-ethyl-4-methoxy-5-methylpyran-2-one (**C_72_**), arenol A (**C_77_**), homoarenol (**C_78_**), arzanol (**C_80_**), 22,22-dimethylitalipyron (**C_84_**), methylarzanol (**C_86_**), and arenol C (**C_87_**). These compounds eluted in the late part of the chromatographic run, between t_R_ 81.2 and 89.6 min, indicating their relatively lipophilic character. Negative-ion MS^2^ and MS^3^ spectra of the selected α-pyranones with proposed structures of the selected most abundant product ions are presented in Figure S7.

The distribution of α-pyranone-type compounds differed among the analyzed extracts. **C_72_** was the most widely distributed compound from this group and was detected in the mother plant extract, all three plantlet extracts, and all three callus extracts, but not in the inflorescence extract. Arenol A was detected in Ha-I, Ha-P-1, and Ha-P-12, whereas homoarenol was present only in Ha-I. Arzanol occurred in Ha-I, Ha-MP, Ha-P-1, and Ha-P-4, while 22,22-dimethylitalipyron was detected in Ha-I and in all three plantlet lines. Methylarzanol was restricted to plantlet extracts. Arenol C was detected only in Ha-I and Ha-P-12.

The late elution of these compounds confirmed their relatively lipophilic character. Their distribution showed that α-pyrone-type metabolites were not limited to inflorescences, as several derivatives were also present in plantlet extracts. The detection of **C_72_** in all callus lines suggests that at least one α-pyrone-related pathway remained active in undifferentiated tissue, whereas the absence of the remaining α-pyrone derivatives from callus extracts indicates that this profile was only partially retained.

##### Phthalides

Phthalides are a group of lactones based on the 1(3H)-isobenzofuranone scaffold. Natural phthalides occur in several plant groups and have been reviewed as structurally diverse specialized metabolites, including monomeric and dimeric derivatives, with reported biological activities such as anti-inflammatory, antimicrobial, cytotoxic, neuroprotective, and cardiovascular effects [42,43]. Although they are less abundant and less frequently discussed than flavonoids or phenolic acids, phthalide-type compounds have also been reported among the specialized metabolites of *H. arenarium*. Previous phytochemical studies summarized for this species described several phthalides and related glycosides in *H. arenarium* inflorescences, including isobenzofuranone-type derivatives [2].

In the present study, one phthalide-type compound was identified, namely 7-*O*-glucoxy-5-hydroxy-1(3H)-isobenzofuranone (**C_57_**). This compound was detected at t_R_ 66.2 min and showed a deprotonated molecular ion at *m*/*z* 327. Negative-ion MS^2^ spectrum of the compound with proposed structures of selected product ions is shown in Figure S8. The distribution of **C_57_** was limited to selected non-inflorescence samples. It was detected in the mother plant extract Ha-MP, in two in vitro-derived plantlet extracts, Ha-P-1 and Ha-P-4, and in one callus extract, Ha-C-12. In contrast, this compound was not observed in the inflorescence extract Ha-I, in plantlet line Ha-P-12, or in callus lines Ha-C-1 and Ha-C-4.

Its presence in both plantlets and one callus line suggests that at least part of the phthalide-related metabolic capacity may be retained under in vitro culture conditions. However, because only one compound from this class was detected, phthalides represented a minor and selectively distributed fraction of the overall *H. arenarium* metabolite profile in the present study.

##### Everlastosides, Spiroketals, and Other Specialized Metabolites

In addition to the major classes discussed above, several other specialized metabolites were detected in the analyzed extracts. These compounds represented structurally diverse groups, including aromatic glycosides, spiroketal-type compounds, and other oxygenated lipophilic metabolites. Although they were less systematically represented than phenolic acids or flavonoids, such compounds have previously been reported as part of the broader phytochemical diversity of *H. arenarium*, particularly among glycosides of aromatic compounds and related specialized metabolites [2].

Aromatic glycosides were represented by three everlastosides: everlastoside E (**C_16_**), everlastoside D (**C_21_**), and everlastoside A (**C_27_**). Everlastosides A-E were previously isolated from the flowers of *H. arenarium*, supporting the relevance of this subgroup for the species [5]. In the present study, everlastoside E was detected at t_R_ 24.9 min and showed [M−H]^−^ at *m*/*z* 431 (Figure S9), with fragment ions at *m*/*z* 385, 369, 329, 243, 203, and 125. Everlastoside D showed [M−H]^−^ at *m*/*z* 393 and fragment ions at *m*/*z* 311 and 149 (Figure S9), whereas everlastoside A showed [M−H]^−^ at *m*/*z* 401 and fragment ions at *m*/*z* 383, 357, 261, 221, 195, and 177 (Figure S10). These compounds were distributed mainly in *ex vitro* material and selected plantlet lines, indicating that the production of everlastoside-type aromatic glycosides was associated with differentiated tissues rather than callus cultures.

Two spiroketal-type compounds were also detected: helispiroketal G (**C_69_**) and helispiroketal C (**C_88_**). Helispiroketal G was detected at t_R_ 80.1 min and showed [M−H]^−^ at *m*/*z* 453 with a product ion at *m*/*z* 255, whereas helispiroketal C was detected at t_R_ 90.0 min and showed [M−H]^−^ at *m*/*z* 399, with fragment ions at *m*/*z* 330 and 287. Chemical structures and negative-ion MS^2^ spectra of compounds **C_69_** and **C_88_** are shown in Figure S11. Notably, the contrasting distribution of these two compounds points to biomass-dependent accumulation of spiroketal-type metabolites. Helispiroketal G was detected exclusively in callus cultures, whereas helispiroketal C occurred in the inflorescence extract and selected plantlet extracts.

The remaining compounds included 6-hydroxy-7-methoxy-2-(2-phenylethyl)chromone (**C_71_**), fargesone A (**C_74_**), fargesone B (**C_79_**), petiolactone A (**C_81_**), and obtusifolin trimethyl ether (**C_85_**). Negative-ion MS^2^ and MS^3^ spectra of the selected compounds are shown in Figure S12. Compound **C_71_** was broadly distributed and was detected in all analyzed extracts, suggesting that this chromone-type metabolite is a common component of the investigated *H. arenarium* materials. Fargesone A and fargesone B were detected in selected differentiated samples, while petiolactone A occurred in several ex vitro and plantlet extracts. **C_85_** was detected only in the inflorescence extract.

Overall, this group illustrates the chemical heterogeneity of *H. arenarium* metabolites. The presence of everlastosides confirms the occurrence of aromatic glycosides previously described for the species, whereas the detection of helispiroketals and other more lipophilic metabolites expands the qualitative profile of the analyzed extracts. Their uneven distribution among inflorescences, mother plants, plantlets, and callus cultures further suggests that tissue differentiation and in vitro culture conditions affect the phytochemical profile of the plant material, which also should be considered during assessment of botanicals’ bioactive potential.

##### Saccharides

Saccharides are primary plant metabolites involved in carbon storage, transport, and osmotic regulation. Although they are not specific chemotaxonomic markers, their detection may provide information on the metabolic status of the analyzed material [2]. In the present study, three saccharide-type compounds were detected: saccharose/sucrose (**C_3_**), raffinose/melitose (**C_4_**), and isomaltotriose (**C_5_**). All of them eluted at the beginning of the chromatographic run, consistent with their high polarity. Saccharose was detected at t_R_ 1.6 min and showed [M−H]^−^ at *m*/*z* 341. Raffinose and isomaltotriose were detected at t_R_ 1.7 and 1.9 min, respectively, both with [M−H]^−^ at *m*/*z* 503, but with different MS/MS fragmentation patterns.

Saccharose was detected in all analyzed extracts. In contrast, raffinose and isomaltotriose were detected only in callus tissue extracts Ha-C-1, Ha-C-4, and Ha-C-12.The sugar profile likely reflects the physiological status of the analyzed biomass rather than species-specific specialized metabolism. While sucrose was ubiquitous, raffinose/melitose and isomaltotriose were restricted to callus cultures, which may be related to the carbohydrate metabolism of rapidly proliferating undifferentiated tissue.

Overall, the phytochemical profile of *H. arenarium* strongly depended on the type of plant material. Metabolites found in inflorescences were dominated by typical flower-associated flavonoids, especially chalcones, flavanones, flavones, and kaempferol derivatives. In contrast, callus cultures displayed a simplified flavonoid profile but retained strong phenolic acid metabolism, particularly caffeoylquinic and dicaffeoylquinic acid derivatives, but also helispiroketal G and several unidentified compounds.

Plantlets showed an intermediate profile, retaining selected flavones, methylated flavonols, α-pyrones, and minor specialized metabolites. These results demonstrate that in vitro-derived biomass does not chemically reproduce the inflorescence profile, but represents a metabolically distinct material with potential value as a source of selected phenolic acids and other specialized metabolites.

#### 2.2.2. Quantitative Analysis of Selected Compounds

**Figure 7:**
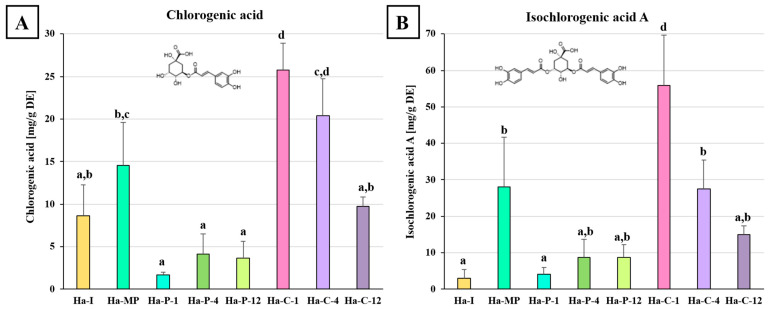
Content of selected phenolic acids in *H. arenarium* extracts. (**A**) Chlorogenic acid and (**B**) isochlorogenic acid A content in extracts obtained from inflorescences (Ha-I), shoots of the mother plant (Ha-MP), in vitro-derived plantlets (Ha-P-1, Ha-P-4, Ha-P-12), and callus cultures (Ha-C-1, Ha-C-4, Ha-C-12). Results are expressed as mg/g of dry extract (DE) and presented as mean ± SD (*n* = 3). Different letters above the bars indicate statistically significant differences between groups according to one-way ANOVA followed by Tukey’s HSD post hoc test (*p* < 0.05).

Quantitative UHPLC-DAD analysis revealed marked differences in the accumulation of chlorogenic acid and isochlorogenic acid A in the investigated *H. arenarium* extracts (Figure 7). Chlorogenic acid was detected in all analyzed samples, but its content strongly depended on the source of plant material. The highest level was observed in the callus extract Ha-C-1 (25.80 ± 3.09 mg/g DE), followed by Ha-C-4 (20.38 ± 4.35 mg/g DE). Both callus lines accumulated significantly higher amounts of chlorogenic acid than most extracts obtained from in vitro-derived plantlets. In contrast, Ha-P-1, Ha-P-4 and Ha-P-12 contained relatively low levels of this compound, ranging from 1.65 to 4.15 mg/g DE. The inflorescence extract Ha-I showed an intermediate chlorogenic acid content (8.64 ± 3.64 mg/g DE), while the mother plant shoot extract Ha-MP contained 14.52 ± 5.12 mg/g DE.

A similar, but even more pronounced, pattern was observed for isochlorogenic acid A. The highest content was found in Ha-C-1 (55.83 ± 13.78 mg/g DE), which was significantly higher than in all other samples. Ha-MP and Ha-C-4 also contained relatively high levels of isochlorogenic acid A, reaching 28.13 ± 13.63 and 27.57 ± 7.93 mg/g DE, respectively. In contrast, Ha-I and Ha-P-1 were characterized by the lowest contents of this metabolite, 3.00 ± 2.38 and 4.16 ± 1.70 mg/g DE, respectively. Ha-P-4, Ha-P-12 and Ha-C-12 showed intermediate values and did not differ significantly from both the low- and medium-accumulating groups according to Tukey’s HSD test.

Free apigenin was additionally quantified using its corresponding reference standard. Apigenin was detected only in the ex vitro-derived samples. In the inflorescence extract Ha-I, its content reached 3.55 ± 1.42 mg/g DE, whereas in the mother plant shoot extract Ha-MP it was detected below the limit of quantification. In the analyzed in vitro-derived plantlets and callus cultures, free apigenin was not detected at quantifiable levels. This indicates that the accumulation of the aglycone form of apigenin was mainly associated with inflorescence material. However, this does not exclude the presence of apigenin-related conjugates in selected samples, as indicated by qualitative UHPLC-DAD-ESI-MS^3^ profiling.

These results indicate that the accumulation of caffeoylquinic acid derivatives in *H. arenarium* is strongly dependent on the type of biomass and culture line. The particularly high levels of both chlorogenic acid and isochlorogenic acid A in selected callus extracts, especially Ha-C-1, suggest that callus cultures may represent a valuable source of these phenolic acids. The observed differences between plantlet and callus lines also show that in vitro culture cannot be treated as a uniform source of phytochemicals. Although all plantlet-derived extracts originated from the same species and were maintained under controlled conditions, their contents of chlorogenic acid and isochlorogenic acid A remained relatively low compared with the most productive callus lines. This suggests that dedifferentiated callus tissue may redirect phenolic metabolism toward the accumulation of caffeoylquinic acid derivatives, but this effect is strongly line-dependent. In contrast, the quantifiable accumulation of free apigenin only in Ha-I points to a stronger association of this aglycone with differentiated inflorescence tissue. Such variability highlights the need for chemical screening and line selection when developing *H. arenarium* in vitro cultures as a source of bioactive metabolites.

The high content of investigated metabolites in selected *H. arenarium* extracts is consistent with previous quantitative studies on this species. Babotă et al. [6] reported chlorogenic acid in *H. arenarium* flowers at 177.70 ± 8.88, 111.58 ± 5.57 and 340.95 ± 17.04 mg/100 g DW in methanolic, ethanolic and 70% ethanolic extracts, respectively. A broad review of *H. arenarium* phytochemistry also indicated substantial variability in chlorogenic acid content in *Helichrysi flos*, ranging from 4.5 to 1700 mg/100 g, depending on subspecies, population and material origin [2]. In this context, our results confirm that chlorogenic acid is quantitatively relevant in *H. arenarium*, but additionally show that selected in vitro-derived callus lines, especially Ha-C-1 and Ha-C-4, may accumulate this marker at particularly high levels when expressed per dry extract. Babotă et al. [6] also reported free apigenin in *H. arenarium* flowers at approximately 63–70 mg/100 g DW, depending on the extraction solvent and hydrolysis step. Quantitative data for isochlorogenic acid A in *H. arenarium* are much more limited; therefore, the high level observed in Ha-C-1 highlights this callus line as a promising and previously poorly characterized source of dicaffeoylquinic acid derivatives.

#### 2.2.3. Total Phenolic and Flavonoid Content and Antioxidant Power of Ex Vitro- and in Vitro-Derived Biomass

**Figure 8:**
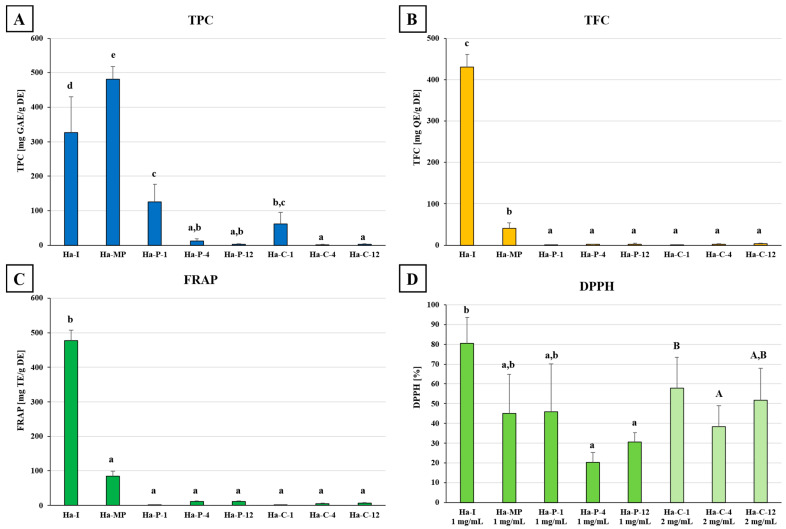
Total phenolic and flavonoid contents and antioxidant activity of methanolic *H. arenarium* extracts obtained from inflorescences (Ha-I), aerial parts of the mother plant (Ha-MP), in vitro-derived plantlets (Ha-P-1, Ha-P-4, and Ha-P-12), and callus cultures (Ha-C-1, Ha-C-4, and Ha-C-12). (**A**) Total phenolic content (TPC), expressed as mg of gallic acid equivalents per g of dry extract (mg GAE/g DE); (**B**) total flavonoid content (TFC), expressed as mg of quercetin equivalents per g of dry extract (mg QE/g DE); (**C**) ferric-reducing antioxidant power (FRAP), expressed as mg of Trolox equivalents per g of dry extract (mg TE/g DE); and (**D**) DPPH radical-scavenging activity, expressed as percentage inhibition. Data are presented as mean ± SD (*n* = 9). In panels (**A**–**C**), different lowercase letters indicate statistically significant differences among extracts within each panel. In panel (**D**), statistical comparisons were performed separately for extracts tested at 1 mg/mL and 2 mg/mL. Statistical significance was assessed using one-way ANOVA followed by Tukey’s HSD post hoc test (*p* < 0.05).

The present results showed clear differences between ex vitro-derived and in vitro-derived *H. arenarium* biomass in terms of phytochemical composition, total phenolic content, total flavonoid content, and antioxidant potential. The highest TPC value was observed in extracts obtained from mother plant shoots, reaching 481.6 ± 37.01 mg GAE/g DE. In contrast, the highest TFC value was recorded in inflorescence extracts, reaching 430.78 ± 30.69 mg QE/g DE. The maximum TPC and TFC values in inflorescence and mother plant shoot extracts reached 326.7 ± 104.39 mg GAE/g DE and 40.58 ± 13.84 mg QE/g DE, respectively. Whereas plantlets and callus cultures generally contained lower total amounts of phenolics and flavonoids. Among plant material derived from in vitro cultures, both plantlets and callus of line 1 produced the highest phenolic quantities up to 125. 4 ± 50.41 mg GAE/g DE and 62.22 ± 32.69 mg GAE/g DE, respectively (Figure 8). These results corroborate the results of UHPLC-DAD-ESI-MS^3^ profiling and quantitative analysis (Table 1; Figure 7), which demonstrated the highest chlorogenic and isochlorogenic A acid concentrations in Ha-C-1 callus extracts.

This pattern is consistent with the known phytochemistry of *H. arenarium*, whose inflorescences are particularly rich in phenolic compounds, especially flavonoids, chalcones, flavanones, flavones, flavonols and phenolic acids [2,3,6,7]. The predominance of phenolic compounds in differentiated flower material has also been reported in previous studies, in which *H. arenarium* flowers contained characteristic constituents such as isosalipurposide, naringenin and its glycosides, apigenin, quercetin and kaempferol derivatives [2,3,6].

The FRAP assay revealed a markedly higher reducing capacity of the inflorescence extract (Ha-I), which reached approximately 480 mg TE/g DE and was significantly more active than all other extracts. The mother plant extract (Ha-MP) showed a considerably lower FRAP value of about 80 mg TE/g DE, whereas the in vitro-derived plantlet and callus extracts exhibited only weak reducing activity and did not differ significantly from one another.

In the DPPH assay, statistical comparisons were performed separately for extracts tested at 1 and 2 mg/mL. At 1 mg/mL, Ha-I showed the strongest radical-scavenging activity, with approximately 80% inhibition, and was significantly more active than Ha-P-4 and Ha-P-12. Ha-MP and Ha-P-1 displayed intermediate activity of approximately 45–46% and did not differ significantly from either Ha-I or the less active plantlet extracts. Among the callus extracts tested at 2 mg/mL, Ha-C-1 exhibited the highest DPPH inhibition, approximately 58%, and was significantly more active than Ha-C-4, which showed about 38% inhibition. Ha-C-12 displayed an intermediate response of approximately 52% and did not differ significantly from either Ha-C-1 or Ha-C-4.

The lower TPC and TFC values in the in vitro-derived samples may therefore result from the absence of full tissue differentiation and flower-specific metabolic regulation. In the present UHPLC-DAD-ESI-MS^3^ profiling, isosalipurposide and most naringenin-derived flavanones were detected only in ex vitro material, particularly in the inflorescences. This indicates that the biosynthesis of these typical flower-associated flavonoids was not efficiently reproduced under the applied in vitro culture conditions. Similar differences between plant organs, developmental stages and extraction conditions have been emphasized for *Helichrysum* species, where chemical composition and biological activity depend strongly on plant material, solvent and preparation method [7,44,45].

However, the in vitro-derived biomass should not be regarded as phytochemically inactive. Although callus cultures showed a simplified flavonoid profile, selected callus lines, especially Ha-C-1 and Ha-C-4, accumulated high amounts of chlorogenic acid and isochlorogenic acid A. This suggests a metabolic shift from flower-associated chalcone/flavanone biosynthesis toward hydroxycinnamic acid and caffeoylquinic acid derivative production. Such line-dependent variability is typical for plant tissue cultures, where dedifferentiation, plant growth regulators, stress conditions, and culture-line selection may redirect secondary metabolism. Therefore, *H. arenarium* callus cultures may represent a promising renewable source of selected phenolic acids rather than a direct chemical equivalent of the traditionally used inflorescences.

The antioxidant results can also be interpreted in relation to this phytochemical pattern. Phenolic acids and flavonoids are known to act as hydrogen- or electron-donating antioxidants, and previous studies on *H. arenarium* demonstrated radical-scavenging and reducing activities associated with polyphenolic constituents. Babotă et al. reported that antioxidant activity of *H. arenarium* flower extracts was related to phenolic composition, with 70% ethanol extracts showing particularly effective DPPH activity [6]. Judžentienė et al. also showed that methanolic extracts of *H. arenarium*, rich in luteolin-7-*O*-glucoside, naringenin derivatives, apigenin, chlorogenic acid, arenol and arzanol, had stronger radical-scavenging activity than essential oils and that antioxidant activity correlated with total polyphenolic content [8].

Nevertheless, the relationship between TPC/TFC and antioxidant power is not always strictly proportional. DPPH and FRAP assays reflect different antioxidant mechanisms: DPPH mainly evaluates radical-scavenging capacity, whereas FRAP measures ferric-reducing power. Therefore, extracts enriched in caffeoylquinic and dicaffeoylquinic acid derivatives may retain relevant antioxidant potential even if their total flavonoid content is lower. This may explain why selected callus extracts, despite lower overall TFC and the absence of several flower-specific flavonoids, still displayed measurable antioxidant activity. Similar observations were made for *H. italicum*, where total phenolic content did not always translate proportionally into DPPH activity, indicating that qualitative composition and the presence of specific redox-active compounds are decisive for antioxidant performance [44].

Overall, the results indicate that ex vitro plant material, especially inflorescences and mother plant shoots, remains the richest source of total phenolics and flavonoids, which is in agreement with the traditional use of *H. arenarium* flowers. In contrast, in vitro-derived biomass differs qualitatively and quantitatively from flower material, but selected callus lines may be valuable for the production of chlorogenic and isochlorogenic acids. Further optimization by line selection, elicitation, light manipulation, or modification of plant growth regulators may enhance the accumulation of target phenolics and improve the antioxidant potential of *H. arenarium* cultures.

### 2.3. Collagenase and Hyaluronidase Inhibitory Activity of H. arenarium Extracts

**Figure 9:**
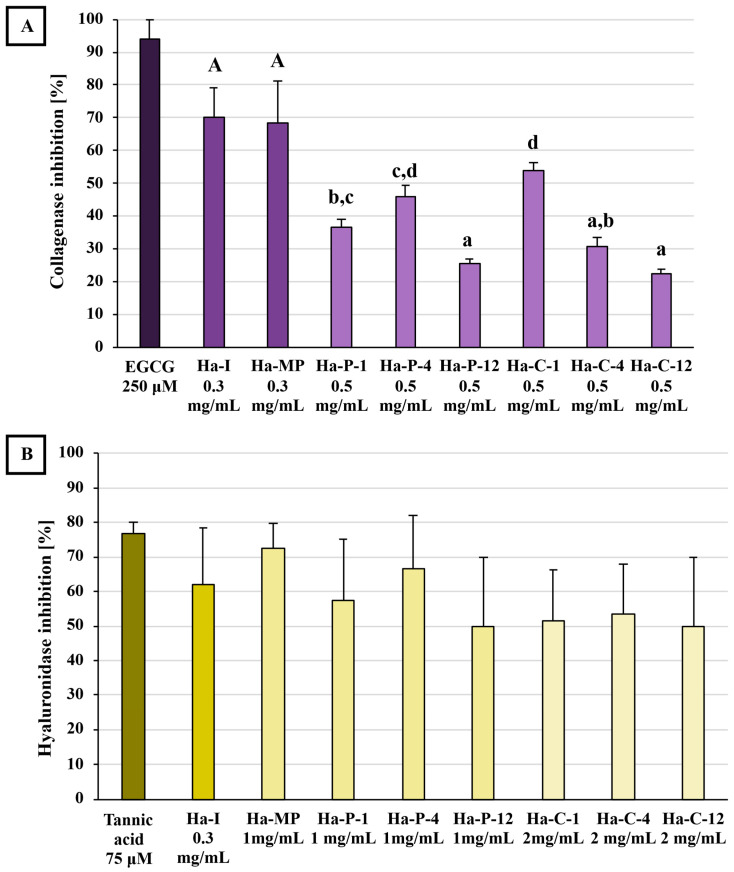
Inhibitory effects of methanolic *H. arenarium* extracts obtained from inflorescences (Ha-I), aerial parts of the mother plant (Ha-MP), in vitro-derived plantlets (Ha-P-1, Ha-P-4, and Ha-P-12), and callus cultures (Ha-C-1, Ha-C-4, and Ha-C-12) on the activities of collagenase (**A**) and hyaluronidase (**B**). The concentrations of the individual extracts are indicated below the corresponding bars. Epigallocatechin gallate (EGCG; 250 µM) and tannic acid (75 µM) were used as positive controls in the collagenase and hyaluronidase assays, respectively. Data are presented as mean ± SD (*n* = 3). Statistical comparisons were performed only among extracts tested at the same concentration. Groups sharing at least one letter are not significantly different, whereas groups with no letters in common differ significantly according to one-way ANOVA followed by Tukey’s HSD post hoc test (*p* < 0.05). In panel (**B**), no statistically significant differences were detected among extracts tested at the same concentration.

The tested *H. arenarium* extracts inhibited both hyaluronidase and collagenase activity, with clear differences between the analyzed biomass types. In the collagenase assay, EGCG inhibited enzyme activity by 94.00 ± 5.70%. The most active *H. arenarium* extracts were Ha-I and Ha-MP, with 70.05 ± 8.98% and 68.40 ± 12.86% inhibition, respectively. Among the in vitro-derived samples, Ha-C-1 showed the highest collagenase inhibition (53.89 ± 2.29%), followed by Ha-P-4 (45.98 ± 3.42%). The other extracts displayed lower activity, ranging from 22.32 to 36.61% (Figure 9A).

In the hyaluronidase assay, tannic acid used as the positive control showed 76.63 ± 3.32% inhibition. Among the extracts, the strongest activity was observed for Ha-MP (72.55 ± 7.08%), followed by Ha-P-4 (66.53 ± 15.49%) and Ha-I (62.00 ± 16.39%). The remaining samples also showed moderate activity, with inhibition ranging from 49.73 to 57.37% (Figure 9B).

These results are consistent with the phytochemical and antioxidant data obtained for the same extracts. The highest TPC and TFC were recorded in the ex vitro-derived material, especially Ha-MP and Ha-I. Ha-MP showed the highest TPC value, 481.6 ± 37.01 mg GAE/g DE, followed by Ha-I, 326.7 ± 104.39 mg GAE/g DE. In contrast, plantlets and callus cultures contained lower total phenolic levels, although Ha-C-1 was distinguished by high chlorogenic acid and isochlorogenic acid A content.

The stronger collagenase inhibition observed for Ha-I and Ha-MP corresponds with their richer flavonoid profile. In the phytochemical analysis, isosalipurposide was detected only in Ha-I and Ha-MP, while naringenin-derived flavanones were restricted to ex vitro-derived material. Ha-I also contained the most diverse flavanone profile. This agrees with published data showing that *H. arenarium* flowers are rich in flavonoids, particularly flavanones such as naringenin derivatives, as well as apigenin derivatives and other phenolic constituents [45].

The biological relevance of collagenase and hyaluronidase inhibition is supported by previous anti-aging studies. Collagen is the main structural protein responsible for dermal strength, whereas hyaluronic acid contributes to hydration and elasticity. Collagenase and hyaluronidase participate in the degradation of these extracellular matrix components, and their inhibition is commonly used as an in vitro indicator of anti-aging potential [46,47].

The obtained results are also in line with reports showing that plant extracts rich in phenolic compounds may display anti-collagenase, anti-elastase or anti-hyaluronidase activity. Thring et al. demonstrated that several phenolic-rich plant extracts inhibited collagenase and elastase, with white tea showing the strongest activity and the highest phenolic content [47]. Jiratchayamaethasakul et al. [46] also showed that plant extracts may differ in their activity against individual skin-aging-related enzymes, with *Rosa rugosa* showing the highest anti-collagenase activity and *Salicornia europaea* the strongest anti-hyaluronidase and anti-elastase activity among the tested halophytes.

### 2.4. Effects of H. Arenarium Extracts in HaCaT Keratinocytes

#### 2.4.1. Cytocompatibility of *H. Arenarium* Extracts in HaCaT Keratinocytes

**Figure 10:**
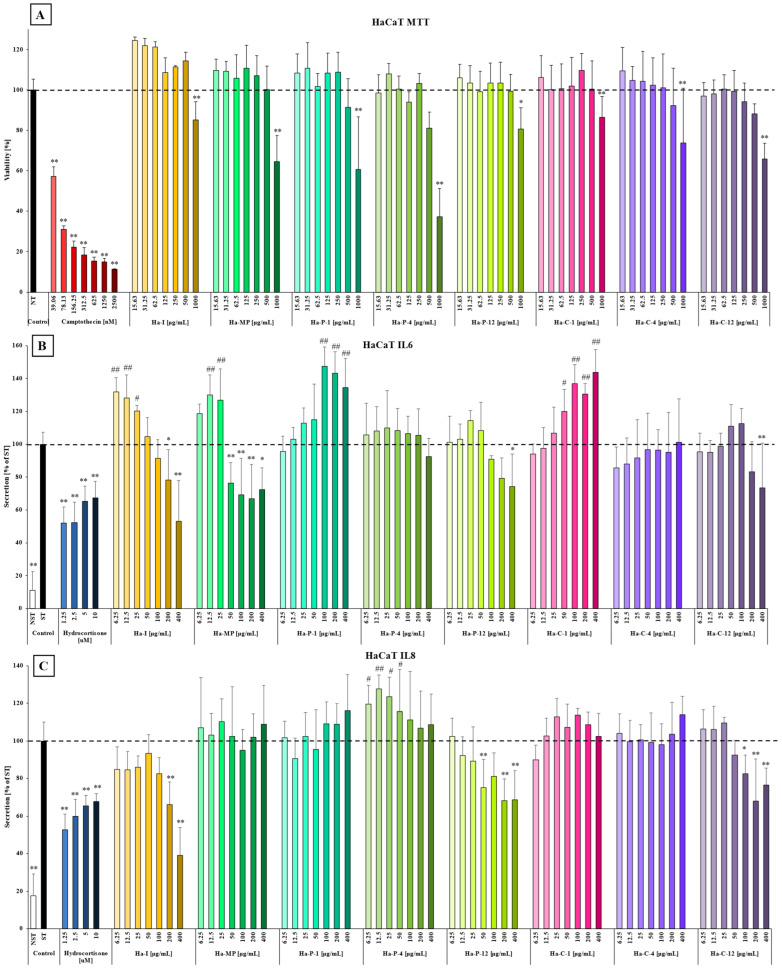
Effect of *H. arenarium* extracts on HaCaT cell viability and pro-inflammatory cytokine secretion. (**A**) Cell viability assessed by the MTT assay after treatment with *H. arenarium* extracts and camptothecin, expressed as a percentage of the non-treated control (NT). (**B**,**C**) Secretion of IL-6 (B) and IL-8 (C) by TNF-α/IFN-γ-stimulated HaCaT cells after treatment with hydrocortisone or *H. arenarium* extracts, expressed as a percentage of the stimulated control (ST). Ha-I, inflorescence extract; Ha-MP, mother plant extract; Ha-P-1, Ha-P-4 and Ha-P-12, extracts from in vitro-derived plantlets; Ha-C-1, Ha-C-4 and Ha-C-12, extracts from callus cultures. Data are presented as mean ± SD (*n* = 3). For the MTT assay, asterisks indicate values significantly lower than the NT control. For IL-6 and IL-8 secretion, asterisks indicate values significantly lower than ST, whereas hash symbols indicate values significantly higher than ST. Statistical significance was assessed using one-way ANOVA followed by Dunnett’s post hoc test: *, # *p* < 0.05; **, ## *p* < 0.001.

The cytocompatibility of *H. arenarium* extracts was first assessed in HaCaT keratinocytes using the MTT assay (Figure 10A). Camptothecin, used as a cytotoxic reference compound, significantly reduced cell viability across the tested concentration range (39.06–2500 nM), confirming the responsiveness of the assay. In contrast, most *H. arenarium* extracts did not markedly affect HaCaT viability at concentrations (15.63–500 μg/mL) corresponding to those used in the cytokine secretion experiments (6.25–400 μg/mL). A significant decrease in cell viability was observed mainly at the highest tested concentration, particularly for Ha-I, Ha-MP, Ha-P-1, Ha-P-4, Ha-P-12, Ha-C-4 and Ha-C-12 at 1000 µg/mL. Since the ELISA experiments were performed at lower concentrations, these results indicate that the observed changes in cytokine secretion were not primarily driven by a nonspecific loss of viable cells.

Only limited data are available regarding the cytocompatibility of *H. arenarium* in skin-related cellular models. Park et al. [48] investigated *H. arenarium* extract in blue light-irradiated HaCaT keratinocytes using concentrations of 0.01–0.1% and showed that the extract counteracted the blue light-induced reduction in cell proliferation. More recent cytotoxicity studies were performed mainly in non-skin or cancer-related models. For example, Aricioglu et al. [49] treated ECV304 and Ishikawa cells with 5—500 µg/mL of *H. arenarium* DMSO extract for 24 h and observed reduced viability at 500 µg/mL, while Nalkiran and Sevim Nalkiran [50] tested *H. arenarium* ethanol and methanol extracts at 0–2.5 µg/mL for 24 and 48 h, demonstrating a cell type-dependent cytotoxic effect that was particularly evident in breast and bladder cancer cells, whereas non-cancerous cells were only minimally affected. Compared with these reports, our study demonstrates that most *H. arenarium* extracts were largely cytocompatible with HaCaT keratinocytes over a relatively broad concentration range of 15.63–500 µg/mL, with significant viability reduction occurring mainly at 1000 µg/mL.

Importantly, to our knowledge, this is the first comparative study providing in vitro evidence for the cytocompatibility of *H. arenarium* extracts obtained from inflorescences, mother plant shoots, in vitro-derived plantlets, and callus cultures in a human keratinocyte model. These results support the potential safety of selected *H. arenarium*-derived extracts for skin-related applications within an appropriate concentration range and provide a necessary basis for interpreting their cytokine-modulating effects.

#### 2.4.2. Modulation of IL-6 and IL-8 Secretion in TNF-α/IFN-γ-Stimulated HaCaT Keratinocytes

The anti-inflammatory potential of the extracts was then evaluated in TNF-α/IFN-γ-stimulated HaCaT keratinocytes by measuring IL-6 and IL-8 secretion (Figure 10B,C). The stimulated control (ST) showed increased cytokine release compared with the non-stimulated control (NST), confirming the induction of an inflammatory-like response. Hydrocortisone (1.25–10 μM) significantly reduced both IL-6 and IL-8 secretion at all tested concentrations, validating the experimental model and providing a reference anti-inflammatory response.

In the IL-6 assay, the activity of the extracts was strongly dependent on the type of plant material and concentration (Figure 10B). Ha-I significantly reduced IL-6 secretion at 200 and 400 µg/mL, whereas its lower concentrations increased IL-6 release relative to ST. A similar biphasic response was observed for Ha-MP, which decreased IL-6 secretion at 50–400 µg/mL but increased it at 12.5 and 25 µg/mL. Among the in vitro-derived materials, Ha-P-12 and Ha-C-12 reduced IL-6 secretion only at the highest tested concentration of 400 µg/mL. In contrast, Ha-P-1 and Ha-C-1 enhanced IL-6 secretion at selected concentrations, indicating that not all in vitro-derived extracts displayed an anti-inflammatory-like profile.

The IL-8 response showed a partially different pattern (Figure 10C). Ha-I significantly reduced IL-8 secretion at 200 and 400 µg/mL, confirming the inhibitory effect of the inflorescence extract at higher concentrations. Among the in vitro-derived extracts, the most pronounced IL-8-lowering activity was observed for Ha-P-12 and Ha-C-12. Ha-P-12 significantly reduced IL-8 secretion at 50, 200 and 400 µg/mL, while Ha-C-12 was active at 100, 200 and 400 µg/mL. Conversely, Ha-P-4 increased IL-8 secretion at 6.25–50 µg/mL, suggesting a pro-inflammatory-like effect within this concentration range. The remaining extracts were mostly neutral in the IL-8 assay.

The increases in IL-6 or IL-8 secretion observed at selected lower extract concentrations, followed by inhibitory effects at higher concentrations, indicate a non-monotonic concentration-dependent response. Such responses may arise from the overlapping effects of multiple biological processes with different concentration dependencies, which is particularly plausible for chemically complex botanical extracts. Previous studies in TNF-α/IFN-γ-stimulated HaCaT keratinocytes have likewise demonstrated that the modulation of inflammatory mediators by plant extracts and their constituents depends strongly on concentration and phytochemical composition [51,52,53]. The mechanisms underlying the cytokine increases observed in the present study were not investigated and require further examination.

Taken together, the cytokine results indicate that *H. arenarium* extracts did not produce a uniform anti-inflammatory response in HaCaT keratinocytes. Instead, their activity was selective and depended on the source of biomass, concentration, and cytokine endpoint. The most promising activity was observed for Ha-I, Ha-P-12, and Ha-C-12. Ha-I reduced both IL-6 and IL-8 at higher concentrations, whereas Ha-P-12 and Ha-C-12 were particularly effective in reducing IL-8 secretion. This is relevant because IL-8/CXCL8 is an important chemokine involved in the recruitment of inflammatory cells to sites of skin inflammation. Therefore, the ability of selected extracts to reduce IL-8 secretion without marked cytotoxicity suggests a potentially beneficial immunomodulatory profile.

The activity of Ha-I is consistent with previous reports describing the anti-inflammatory potential of *H. arenarium* inflorescences. Earlier studies showed that methanolic extracts from *H. arenarium* flowers and selected flavonoid glycosides, including naringenin and apigenin derivatives, may interfere with TNF-α-related inflammatory pathways [5]. Since TNF-α/IFN-γ-stimulated keratinocytes produce IL-6 and IL-8 through inflammatory signaling networks involving, among others, NF-κB and MAPK-related pathways [54,55], the cytokine-lowering activity of selected extracts may be linked to their phenolic and flavonoid constituents. However, this should be regarded as a plausible explanation rather than a confirmed mechanism, as intracellular signaling pathways were not directly investigated in the present study.

The diverse responses observed for extracts obtained from plantlets and callus cultures are particularly important. In vitro cultures alter not only the total level of specialized metabolites but also their relative proportions. As a result, extracts derived from different lines or culture types may differ substantially in biological activity, even when obtained from the same species [56,57]. This may explain why Ha-P-12 and Ha-C-12 reduced cytokine secretion, whereas Ha-P-1, Ha-P-4 or Ha-C-1 increased selected inflammatory mediators. Such findings indicate that the biological value of in vitro-derived *H. arenarium* biomass cannot be assumed solely on the basis of species identity. Instead, each line or culture type requires phytochemical and functional verification.

In conclusion, the biological assays showed that selected *H. arenarium* extracts can modulate inflammatory cytokine secretion in TNF-α/IFN-γ-stimulated HaCaT keratinocytes. The most favorable profiles were observed for Ha-I, Ha-P-12 and Ha-C-12, especially with respect to IL-8 reduction. However, the response was highly dependent on the origin of the plant material and concentration, and some extracts increased cytokine release. These findings support the potential of selected *H. arenarium*-derived materials as sources of anti-inflammatory-like activity, while emphasizing the need for careful selection, phytochemical standardization, and functional screening of in vitro-derived biomass.

#### 2.4.3. Effect of Plant Extracts on Wound Closure in the Scratch Assay

The migratory capacity of HaCaT keratinocytes was evaluated using the in vitro scratch wound-healing assay. Wound closure was assessed at 48 h and expressed as a percentage of the initial scratch area. Overall, the tested extracts exhibited variable, concentration-dependent effects that differed markedly between extracts of tested explants.

Selected extracts significantly enhanced wound closure relative to the untreated control. The Ha-P-1 extract produced the most pronounced pro-migratory response: at 100 µg/mL, wound closure reached 71.01 ± 4.23%, representing about a 96% increase over the control value of 36.38 ± 13.78%. A similarly elevated response was recorded at 200 µg/mL (60.69 ± 7.58%). Comparable stimulatory effects were observed for Ha-P-12 at 100 µg/mL (58.22 ± 16.17%), which represents a 53% more pronounced effect in relation to the control, and for Ha-C-4 extract at 200 µg/mL (47.48 ± 11.88%), which is 56% more enhanced activity as compared to the control. Notably, the inflorescence extracts at 200 µg/mL produced the greatest relative increase across all tested conditions, with wound closure of 43.15 ± 5.56% compared to 18.10 ± 8.66% in controls.

In contrast, the Ha-C-12 extract exhibited a distinct inhibitory profile. At 12.5 µg/mL, wound closure was reduced to 7.99 ± 5.07%, that is, 49.55 ± 22.20% in relation to control, and a similar suppression was recorded at 25 µg/mL (9.66 ± 4.39%). This inhibitory effect was observed at the lowest concentrations tested, suggesting a potent anti-migratory action of this particular extract even at low doses.

The Ha-MP extract did not significantly alter wound closure at any concentration tested, with values remaining close to control levels across the entire concentration range (12.39–16.09% among all tested concentrations and 14.63% in the control). Similarly, the Ha-C-1, Ha-P-4, and Ha-C-4 (at lower concentrations) extracts showed no statistically significant differences from the control, although a slight tendency toward reduced wound closure was observed at 25 µg/mL in Ha-C-1 and Ha-P-4 treatment.

The HaCaT migration results did not directly reflect the overall richness of the *H. arenarium* phytochemical profile. The strongest pro-migratory effect was observed for Ha-P-1 at 100 µg/mL, whereas Ha-MP, despite its richer ex vitro-type profile, did not significantly affect wound closure. Similarly, Ha-I and Ha-MP contained characteristic *H. arenarium* flower-associated metabolites, including isosalipurposide and naringenin-derived flavanones, but these extracts were not the most active in the scratch assay.

This indicates that keratinocyte migration was not determined simply by the presence of typical *H. arenarium* flavanones or by total phenolic/flavonoid richness. Literature data also support this interpretation. *H. arenarium* is known to be rich in flavonoids, especially isosalipurposide, naringenin and naringenin glycosides, while *H. italicum* preparations may contain high amounts of caffeoylquinic acid derivatives and pyrones; however, biological activity differs depending on species, extract type and chemical profile [2,28].

The strongest migration-promoting extract in the present study, Ha-P-1, had an in vitro plantlet-derived profile rather than a typical inflorescence profile. By contrast, callus extracts were characterized by simplified flavonoid composition and higher phenolic acid accumulation, especially in Ha-C-1 and Ha-C-4, but their effects on migration differed: Ha-C-4 stimulated wound closure only at 200 µg/mL, whereas Ha-C-12 inhibited migration at low concentrations.

These findings are consistent with studies on other *Helichrysum* preparations. Serra et al. showed that *H. italicum* hydrolate supported wound-healing-related processes, including collagen deposition, and linked this activity with a profile rich in caffeoylquinic acid and naringenin derivatives [9]. However, in the present *H. arenarium* extracts, the presence of these compound classes alone was not sufficient to predict HaCaT migration.

The results also agree with the work of Skowrońska et al. on *Sambucus nigra* leaves, where ethanolic extracts contained more polyphenols and showed stronger antioxidant and anti-inflammatory activity than aqueous extracts, but the authors emphasized that further cellular models are needed to confirm their wound-healing relevance [58]. They also discussed IL-8 as a mediator that can enhance HaCaT migration and adhesion to the wound site.

Overall, the migration assay suggests an extract-specific response. Ha-P-1 was the most promising for stimulating HaCaT migration, whereas Ha-MP and Ha-I, despite their richer characteristic *H. arenarium* flavonoid profile, were less active in this model. Therefore, the wound-closure effect should be discussed as dependent on the complete chemical profile of each extract rather than on single marker compounds or total phenolic/flavonoid content.

## 3. Materials and Methods

### 3.1. Chemicals and General Procedures

High-glucose Dulbecco’s modified Eagle medium (DMEM) containing L-glutamine and sodium pyruvate, Dulbecco’s phosphate-buffered saline (DPBS) without Ca^2+^ and Mg^2+^, TrypLE Express Enzyme, and Antibiotic-Antimycotic (100×) solution were obtained from Gibco (Thermo Fisher Scientific, Waltham, MA, USA). FBS Superior was purchased from Sigma-Aldrich (Merck KGaA, Darmstadt, Germany). Recombinant human tumor necrosis factor-alpha (TNF-α) and recombinant human interferon-gamma (IFN-γ), supplied under the Gibco PeproTech brand, were obtained from Thermo Fisher Scientific (Waltham, MA, USA). Camptothecin (CPT) and dimethyl sulfoxide (DMSO) were purchased from Sigma-Aldrich (Merck KGaA, Darmstadt, Germany). Collagenase from *Clostridium histolyticum* (ChC; EC 3.4.24.3), Tricine (N-[Tris (hydroxymethyl)methyl]glycine, N-[3-(2-furyl)acryloyl]-Leu-Gly-Pro-Ala (FALGPA), and epigallocatechin gallate were purchased from Sigma-Aldrich (Merck KGaA, Darmstadt, Germany). 96-well Nunc microtitre plates were supplied by Thermo Fisher Scientific, Waltham, MA, USA. 3-*O*-caffeoylquinic acid (chlorogenic acid) and apigenin were purchased from Sigma-Aldrich, St. Louis, MO, USA, while 3,5-di-*O*-caffeoylquinic acid (isochlorogenic acid A) was purchased from PhytoLab, Vestenbergsgreuth, Germany.

The chemicals for in vitro plant cultures: plant agar (P1001), MS medium (M0221), 6-benzylaminopurine (BAP; B0904), 2,4-dichlorophenoxyacetic acid (2,4 D; D0911) were purchased from Duchefa Biochemie B.V. (Haarlem, The Netherlands). Methanol for extraction was delivered by Avantor Performance Materials (Gliwice, Poland).

Thiazolyl blue tetrazolium bromide (MTT) was obtained from Thermo Scientific Chemicals (Thermo Fisher Scientific, Waltham, MA, USA). The BD OptEIA Human IL-6 ELISA Set, BD OptEIA Human IL-8 ELISA Set, and BD OptEIA Reagent Set B were purchased from BD Biosciences (Franklin Lakes, NJ, USA).

Ultrapure water was generated using a Merck Millipore Simplicity UV system. LC-MS-grade solvents used for metabolite extraction and LC-MS analysis, including isopropanol (IPA), methanol (MeOH), acetonitrile (ACN), and water, as well as ammonium formate and formic acid, were purchased from Merck KGaA (Darmstadt, Germany).

### 3.2. Plant Material

Plant material, i.e., 30 aerial parts of *H. arenarium*, were collected with permission of the Regional Director for Environmental Protection in Olsztyn N^o^. WOPN.6400.71.2025.OK.2, from a natural habitat. They were air-dried in darkness. The voucher specimen was deposited in Herbarium Universitatis Varsoviensis N^o^. WA0000169581. The collected seeds were used to establish in vitro cultures.

### 3.3. In Vitro Cultures

The in vitro shoot and callus cultures were established starting from seedlings developed from collected plants as described above. The seeds were soaked for 30 s in 70% (*v*/*v*) ethanol and then sterilized with 2.5% (*w*/*v*) sodium hypochlorite (NaClO) for 15 min and afterwards washed three times with sterile water. Sterile seeds were transferred to 100 mL Erlenmeyer flasks containing 30 mL half-strength MS medium (MS/2; [15]) solidified by addition of 7 g/L of agar, pH = 5.7, adjusted before autoclaving at 1.2 atm. for 20 min. The seeds were incubated in the dark in a culture room at a temperature of 24 ± 2 °C for one week before germination started. Each seed was placed separately in one flask, and resulted 4-week old seedlings gave rise to distinct shoot lines and callus lines. Sixteen of 20 seeds used to initiate *H. arenarium* in vitro cultures germinated and allowed the establishment of 16 shoot line cultures, routinely cultivated on full-strength MS solid medium (50 mL in 300 mL Erlenmeyer flasks) with every 4-week transfer onto fresh medium. For investigations under the conditions of the current study, three shoot lines were chosen. Leaf explants were taken from the shoots of the three lines and used to establish callus cultures on solid MS medium supplemented with 1 mg/L of BAP and 1 mg/L of 2,4-D with every 4-week transfer onto fresh medium. The shoot and callus lines were assigned as follows: (i) shoot lines: Ha-P-1, Ha-P-4, and Ha-P-12; (ii) callus lines: Ha-C-1, Ha-C-4, and Ha-C-12. The shoot and callus cultures were carried out under 12 h/12 h light/dark regime at a temperature of 24 ± 2 °C.

To determine the callus growth rate, expressed as a ratio of final weight to initial weight, of callus tissue, 1.00 ± 0.24 g of 4-week-old callus was placed on the fresh medium and collected after 4 weeks of culture. Next, the final fresh weight was noted, and the dry weight after lyophilisation. The callus biomass increase determination was carried out based on callus tissue collected from at least 6 flasks during three subsequent passages.

### 3.4. Phytochemical Studies

#### 3.4.1. Preparation of Plant Extracts

The extracts subjected to investigations under the conditions of the current study were performed form shoots of maternal plants (Ha-MP), inflorescences (Ha-I), shoots of three lines cultivated in vitro (Ha-P-1; Ha-P-4; Ha-P-12), and three lines of callus tissue (Ha-C-1; Ha-C-4; Ha-C-12). Three samples of maternal plant shoots and three samples of inflorescences were prepared. For each in vitro-derived plantlet and callus line, material from three independent flasks was collected over three consecutive 4-week passages (*n* = 9). Plant material derived in vitro was lyophilised, ground and subjected to extraction. About 1 g of powdered tissue was extracted with 50 mL of 100% methanol, 3 times for 15 min. at an ultrasonic bath (Sonorex Bandelin, Berlin, Germany). Combined supernatants were next evaporated to dryness on a rotary evaporator (Heidolph, Laborota 4000, Schwabach, Germany). The resulting extracts were kept at −20 °C prior to investigations.

#### 3.4.2. Qualitative UHPLC-DAD-ESI-MS^3^ Analysis

The dry extracts (10 mg/mL; DEs) were reconstituted in methanol containing 0.1% (*v*/*v*) formic acid and subjected to qualitative phytochemical profiling. Before injection, the samples were passed through 0.45 µm PVDF syringe filters. The following reference standards were used to support compound identification: 3-*O*-caffeoylquinic acid (chlorogenic acid), apigenin, and 3,5-di-*O*-caffeoylquinic acid (isochlorogenic acid A).

Chromatographic analyses were performed using a UHPLC-3000 RS system (Dionex, Dreieich, Germany) equipped with a diode-array detector (DAD; Dionex) and coupled to an AmaZon SL ion-trap mass spectrometer fitted with an electrospray ionization (ESI) interface (Bruker Daltonik, Bremen, Germany). The compounds were separated on a Kinetex XB-C18 column (150 × 2.1 mm i.d., 1.7 µm; Phenomenex, Torrance, CA, USA) maintained at 25 °C.

The mobile phase consisted of water containing 0.1% (*v*/*v*) formic acid (solvent A) and acetonitrile containing 0.1% (*v*/*v*) formic acid (solvent B). Linear gradient elution was applied as follows: 0–60 min, 1–26% B; and 60–90 min, 26–90% B. The flow rate was set at 0.3 mL min^−1^, and the injection volume was 5 µL. The column was re-equilibrated for 10 min between injections. UV–Vis spectra were recorded over the wavelength range of 200–450 nm.

The eluate was transferred directly to the ESI interface without flow splitting and analyzed in negative-ion mode. The ESI parameters were as follows: nebuliser pressure, 38 psi; dry gas flow rate, 7 L min^−1^; dry gas temperature, 135 °C; and capillary voltage, 4.5 kV. MS^2^ and MS^3^ fragmentation spectra were acquired in Auto MS/MS mode for the most abundant precursor ions detected at a given retention time. Mass spectra were recorded over an *m*/*z* range of 70–2200.

#### 3.4.3. Identification of Metabolites

Metabolites were identified or tentatively assigned based on their molecular masses, MS^2^ and MS^3^ fragmentation patterns, and UV–Vis spectra. The interpretation of the obtained data was supported by literature reports concerning compounds previously detected or isolated from species of the Asteraceae family, with particular emphasis on the genus *Helichrysum*. Potential structural matches were additionally searched in the Reaxys and PubChem databases.

Whenever reference standards were available, compound identities were confirmed by comparison of retention times and spectral characteristics. For compounds with identical molecular masses, the predicted XLogP3-AA values retrieved from PubChem were used as an additional supporting parameter to assess the most probable elution order and facilitate tentative structural assignment.

#### 3.4.4. Quantitative UHPLC-DAD Analysis

Quantitative analysis was performed using the chromatographic data acquired with the DAD detector. The selectivity of the method and the identities of the quantified metabolites were verified based on the previously described UHPLC-DAD-ESI-MS^3^ analysis. The corresponding mass spectra and chromatographic profiles were examined to exclude the co-elution of interfering compounds under the applied analytical conditions.

Selected metabolites were quantified using the corresponding reference standards: chlorogenic acid, isochlorogenic acid A and apigenin. All three compounds were quantified using calibration curves prepared with their respective reference standards. The absorbance of the quantified phenolic acids was monitored at 325 nm, whereas apigenin was monitored at 337 nm according to Kiełkiewicz et al. [59]. Calibration curves were constructed by plotting peak areas against the amounts of the respective standards injected onto the column.

The limits of detection (LOD) and quantification (LOQ) were calculated according to the ICH Q2(R2) guideline using the following equations: LOD = 3.3 σ/S and LOQ = 10 σ/S, where σ is the standard deviation of the response, and S is the slope of the calibration curve. The analytical performance parameters used for comparative quantification, including calibration equations, linearity, working ranges, LOD, and LOQ, are presented in Supplementary Table S1. Values below the LOD were reported as not detected. Signals between the LOD and LOQ were reported as detected but not quantified and were not included as numerical values in the statistical analysis.

#### 3.4.5. Determination of Total Phenolic Content (TPC) and Total Flavonoid

***Content (TFC)***

Total phenolic content (TPC) and total flavonoid content (TFC) of the extracts were determined according to the method developed by Pękal and Pyrzyska [60] with the modifications of Kiełkiewicz et al. [17].

At least three biological replicates, obtained from three consecutive passages, were used for the analysis. For inflorescences and mother plants, three independent samples per material type were analyzed. Each sample was analyzed in triplicate. For all assays, an Epoch BioTek (Agilent, Santa Clara, CA, USA) microplate reader was used.

Total phenolic content (TPC) was determined using the Folin–Ciocalteu colorimetric method adapted to a 96-well microplate format. Dry extracts were dissolved in 80% methanol to obtain a concentration of 10 mg/mL. Briefly, 20 µL of the extract solution was mixed with 40 µL of Folin–Ciocalteu reagent in each well. The plate was shaken for 5 min at 400 rpm and 20 °C. Subsequently, 160 µL of 7% sodium carbonate solution was added, and the plate was shaken for 3 min under the same conditions. The reaction mixture was incubated in the dark for 30 min, and the absorbance was measured at 765 nm using a microplate reader. The results were calculated from a gallic acid calibration curve and expressed as mg of gallic acid equivalents per g of dry extract (mg GAE/g DE).

Total flavonoid content (TFC) was determined using the aluminum chloride colorimetric assay in the presence of sodium nitrite under alkaline conditions, according to Pękal and Pyrzynska [60], with minor modifications for 96-well microplates [59]. Dry extracts were dissolved in 80% methanol to obtain a concentration of 10 mg/mL. Briefly, 50 µL of the extract solution was mixed with 50 µL of 5% NaNO_2_ solution in each well. The plate was shaken for 5 min at 400 rpm and 20 °C. Then, 100 µL of 2% AlCl_3_ solution was added, and the plate was shaken for another 5 min. Finally, 100 µL of 1 M NaOH was added, and after 15 min of shaking, the absorbance was measured at 425 nm. The results were calculated from a quercetin calibration curve and expressed as mg of quercetin equivalents per g of dry extract (mg QE/g DE).

#### 3.4.6. Determination of Antioxidant Activity

To assess the antioxidant potential of plant extracts, the DPPH^•^ radical scavenging and ferric reducing antioxidant power (FRAP) assays were performed.

Antioxidant activity was evaluated using the DPPH radical scavenging assay according to Fukumoto and Mazza [61], with minor modifications. Dry extracts of initial concentration of 10 mg/mL were dissolved in 80% methanol when necessary to ensure measurements within the linear range of the spectrophotometric assay. Before analysis, extracts from inflorescences, mother plants, and in vitro-derived plantlets were diluted tenfold to obtain a working concentration of 1 mg/mL. Due to their lower absorbance, callus extracts were diluted fivefold, yielding a working concentration of 2 mg/mL.

In a 96-well microplate, 20 µL of the extract solution was mixed with 200 µL of DPPH radical solution. The plate was incubated in the dark for 60 min at 20 °C with shaking at 150 rpm. Absorbance was then measured at 520 nm using a microplate reader. Antioxidant capacity was calculated from a Trolox calibration curve and expressed as mg Trolox equivalents per g of dry extract (mg TE/g DE). The radical-scavenging capacity was reported as the percentage of DPPH inhibition and calculated using the following equation:

DPPH inhibition (%) = [(A_0_ − A_s_)/A_0_] × 100

where A_0_ represents the absorbance of the control solution, containing DPPH and 80% methanol, and A_s_ denotes the absorbance measured for the sample.

Ferric reducing antioxidant power (FRAP) was determined according to Benzie and Strain [62], with minor modifications for a 96-well microplate format. Dry extracts were dissolved in 80% methanol to obtain a concentration of 10 mg/mL. Briefly, 10 µL of the extract solution was mixed with 300 µL of freshly prepared FRAP reagent in each well. The plate was incubated in the dark for 30 min at 20 °C with shaking at 150 rpm. The absorbance was measured at 593 nm. The antioxidant capacity was calculated from a Trolox calibration curve and expressed as mg Trolox equivalents per g of dry extract (mg TE/g DE).

### 3.5. Enzyme Inhibitory Assays

#### 3.5.1. Collagenase Inhibitory Activity Assay

The inhibitory activity against collagenase was assessed using a spectrophotometric method based on the procedure described by Van Wart and Steinbrink [63], with modifications adapted for a microplate reader format as described by Thring et al. [47]. All assays were performed in 50 mM Tricine buffer (pH 7.5) supplemented with 400 mM NaCl and 10 mM CaCl_2_.

Collagenase from *Clostridium histolyticum* (ChC; EC 3.4.24.3) was dissolved in Tricine buffer to give an initial stock concentration of 0.8 units/mL, in accordance with the supplier’s activity specifications. The synthetic chromogenic substrate N-[3-(2-furyl)acryloyl]-Leu-Gly-Pro-Ala (FALGPA) was dissolved in Tricine buffer at a concentration of 2 mM. The assay exploits the decrease in absorbance at 335 nm that occurs upon hydrolysis of the furanacryloyl-peptide bond in FALGPA by collagenase.

Prior to the assay, the UV–visible spectra of all plant extracts were recorded on a Synergy 4 microplate reader (BioTek Instruments, Winooski, VT, USA) to verify the absence of spectral interference at 335 nm and to confirm the absence of any shift in the lambda maximum. Due to differences in the concentration of UV–visible absorbing constituents among the extracts, individual samples required different dilution factors before analysis. Dilutions were performed to obtain absorbance values within the linear range of the standard curve and below the maximum acceptable absorbance value (A < 2.5). Extracts obtained from inflorescences and mother plants were diluted 30-fold, resulting in a working concentration of 0.3 mg extract/mL, whereas extracts derived from callus tissue and in vitro-grown plantlets were diluted 20-fold, corresponding to a working concentration of 0.5 mg extract/mL.

Each test extract was pre-incubated with the enzyme solution in Tricine buffer for 30 min at room temperature before initiating the reaction by addition of the substrate. The final reaction mixture (total volume 150 μL) contained 0.8 mM FALGPA, 0.1 units of ChC, and 25 μL of the tested extract dissolved in deionized water. A negative control was prepared by replacing the extract with an equivalent volume of deionised water. Absorbance at 335 nm was recorded immediately after substrate addition and then continuously for 20 min using a Synergy 4 microplate reader (BioTek Instruments, Winooski, VT, USA) in 96-well Nunc microtitre plates (Thermo Fisher Scientific, Waltham, MA, USA). Epigallocatechin gallate (EGCG; concentration in well 250 μM; 0.114 mg/mL) was used as a positive control.

The percentage inhibition of collagenase activity was calculated according to the following equation:

Inhibition (%) = [(OD_control_ − OD_sample_)/OD_control_] × 100

where OD_control_ is the absorbance of the reaction mixture without the test extract, and OD_sample_ is the absorbance of the reaction mixture in the presence of the test extract. The collagenase inhibitory activity of each extract was classified as high (>40% inhibition), moderate (20–40%), or low (<20%), as adapted from Thring et al. [47].

#### 3.5.2. Hyaluronidase Inhibitory Activity Assay

Hyaluronidase inhibitory activity was determined using a modified colorimetric method based on Ding et al. [46]. The assay was performed in 0.1 M acetate buffer (pH 3.6). Because the extracts differed in the levels of compounds absorbing in the UV–visible range, sample-specific dilution factors were applied prior to analysis. The extracts were diluted to ensure that the recorded absorbance values remained within the linear range of the calibration curve and did not exceed the maximum acceptable absorbance of 2.5. Extracts from inflorescences were diluted 30–fold to a final working concentration of 0.3 mg/mL, extracts from mother plants and in vitro-derived plantlets were diluted 10-fold to a final working concentration of 1.0 mg/mL, while callus extracts were diluted 5-fold, yielding a working concentration of 2.0 mg/mL.

Briefly, 25 µL of the tested extract or standard solution was mixed with 20 µL of hyaluronidase (4 mg/mL in acetate buffer) and incubated at 37 °C for 20 min. Subsequently, 40 µL of calcium chloride (12.5 mM) was added, and the mixture was incubated again at 37 °C for 20 min. The reaction was initiated by adding 50 µL of sodium hyaluronate solution (3.5 mg/mL in acetate buffer), followed by incubation at 37 °C for 40 min with shaking. Color development was performed by adding 20 µL of sodium hydroxide (0.9 M) and 40 µL of sodium tetraborate (0.2 M), followed by heating at 100 °C for 30 min. After cooling, 160 µL of p-dimethylaminobenzaldehyde (DMABA) reagent was added, and the mixture was incubated at 37 °C for 20 min. Absorbance was measured at 585 nm. A blank was prepared without hyaluronidase.

The percentage inhibition of hyaluronidase activity was calculated using the following equation:

Inhibition (%) = ((Acontrol − Asample)/Acontrol) × 100

### 3.6. In Vitro Cellular Studies in HaCaT Keratinocytes

HaCaT cells (spontaneously immortalized human epidermal keratinocytes; Cytion GmbH, Heidelberg, Germany) were cultured in high-glucose DMEM containing L-glutamine and sodium pyruvate, supplemented with 10% FBS and 1% Antibiotic-Antimycotic (100×) solution. The cells were maintained in a humidified incubator at 37 °C in an atmosphere containing 5% CO_2_ (Binder, BINDER GmbH, Tuttlingen, Germany).

#### 3.6.1. Evaluation of Cell Viability Using the MTT Assay

The effects of the tested extracts on HaCaT cell viability were assessed using the MTT assay [59]. Cells were seeded in 48-well plates at a density of approximately 1.7 × 10^4^ cells per well and maintained for 48–72 h until they reached 70–80% confluence. Dry extracts obtained from the plant materials described above (Ha-I; Ha-MP; Ha-P-1; Ha-P-4; Ha-P-12; Ha-C-1; Ha-C-4; Ha-C-12) were dissolved in DMSO to prepare stock solutions and diluted in culture medium immediately before the experiment. Camptothecin (CPT) was used as a positive control. The final concentration of DMSO did not exceed 0.25% (*v*/*v*) and was matched in the corresponding non-treated control wells. Preliminary experiments confirmed that this concentration of DMSO did not affect cell viability.

Before treatment, the cells were rinsed with DPBS. The extracts were applied at concentrations ranging from 15.6 to 1000 µg/mL, whereas CPT was tested at concentrations ranging from 39.1 to 2500 nM. Following a 24 h incubation, the treatment medium was removed, and the cells were washed with DPBS. Freshly prepared MTT solution in culture medium was then added to each well at a final concentration of 0.5 mg/mL. After incubation for 1 h, the MTT solution was discarded, and the cells were rinsed with DPBS. The resulting formazan crystals were dissolved in DMSO. The plates were shaken in the dark for 5 min at 400 rpm using a DTS-2 microplate shaker (ELMI Ltd., Riga, Latvia) until complete dissolution of the crystals. Aliquots of 100 µL were transferred to 96-well plates, and absorbance was recorded at 560 nm with a reference wavelength of 620 nm using a Synergy 4 microplate reader (BioTek Instruments, Winooski, VT, USA). Cell viability was calculated as a percentage of the corresponding non-treated control.

#### 3.6.2. Evaluation of Anti-Inflammatory Activity

The effects of the tested extracts on inflammatory mediator secretion were evaluated in cytokine-stimulated HaCaT keratinocytes [64]. Cells were seeded in 24-well plates at a density of approximately 3.3 × 10^4^ cells per well and cultured until they reached approximately 80% confluence. The medium was then removed, the cells were rinsed with DPBS, and fresh culture medium containing the tested extracts was added. The cells were pre-incubated with the extracts at concentrations ranging from 6.25 to 400 µg/mL for 4 h before stimulation. Hydrocortisone (HC; 1.25–10 µM) was used as a positive control for the inhibition of inflammatory mediator secretion.

The inflammatory response was induced by adding a mixture of TNF-α and IFN-γ prepared in DPBS, with each cytokine applied at a final concentration of 10 ng/mL. Cells receiving an equivalent volume of DPBS instead of the cytokine mixture served as the non-stimulated control (NST), whereas cells stimulated with TNF-α and IFN-γ in the absence of the tested extracts served as the stimulated control (ST). Following stimulation, the cells were incubated for 24 h. The culture supernatants were then collected and stored at −20 °C until analysis.

The concentrations of interleukin-6 (IL-6) and interleukin-8 (IL-8/CXCL8) in the collected supernatants were determined using enzyme-linked immunosorbent assays (ELISA) according to the manufacturer’s instructions. The results were expressed as percentages relative to the stimulated control, which was defined as 100%.

#### 3.6.3. Evaluation of Keratinocyte Migration Using the Scratch Assay

HaCaT cells were seeded in 12-well plates at a density of approximately 1.8 × 10^5^ cells per well in 1 mL of complete DMEM and incubated for approximately 24 h until a confluent monolayer was formed. The culture medium was then replaced with serum-free DMEM, and the cells were maintained for an additional 4 h to limit the contribution of cell proliferation. A single linear scratch was subsequently generated across the center of each well using a sterile 200 µL pipette tip [65]. The medium was removed, and the wells were rinsed with DPBS to eliminate detached cells. Serum-free medium alone or serum-free medium containing the tested extracts at concentrations ranging from 6.25 to 200 µg/mL was then added.

Images of the scratched area were captured immediately after treatment application (0 h) and after 48 h of incubation using a Delta Optical IM-100 inverted microscope (Delta Optical Sp. z o.o. Sp.k., Minsk Mazowiecki, Poland).

Keratinocyte migration was assessed based on the reduction in the cell-free scratch area relative to the initial measurement. The degree of scratch closure was calculated by comparing the scratch area recorded at each subsequent time point with the corresponding area measured at 0 h. The Fiji (ImageJ version 1.54t, 64-bit, Windows) was used to elaborate scratch assay results.

### 3.7. Statistical Analysis

The results are presented as mean values ± standard deviation (SD) from at least three independent experiments. For biomass growth, total phenolic content (TPC), total flavonoid content (TFC), and antioxidant activity (DPPH and FRAP assays), nine biological replicates were analyzed per treatment. Cell viability, collagenase- and hyaluronidase-inhibitory activities, wound-healing potential, and anti-inflammatory activity were evaluated using three biological replicates per treatment. Each biological replicate was analyzed in four technical replicates.

Statistical analyses were performed using Statistica, version 13 (TIBCO Software Inc., Palo Alto, CA, USA). For single-factor comparisons, data were analyzed using one-way ANOVA followed by Tukey’s honestly significant difference (HSD) post hoc test. Groups sharing at least one letter were not significantly different, whereas groups with no letters in common differed significantly at *p* < 0.05. For comparisons of individual treatment groups with the corresponding control group, one-way ANOVA followed by Dunnett’s post hoc test was used. Differences were considered statistically significant at *p* < 0.05 and *p* < 0.001. In the MTT assay, asterisks indicate values significantly lower than the non-treated control (NT). For IL-6 and IL-8 secretion, asterisks indicate values significantly lower than the stimulated control (ST), whereas hash symbols indicate values significantly higher than ST: * *p* < 0.05, ** *p* < 0.001, # *p* < 0.05, and ## *p* < 0.001. Hierarchical clustering analysis was performed based on the binary metabolite presence/absence matrix. Percent disagreement was used as the distance measure, and clustering was carried out using the unweighted pair-group method with arithmetic mean (UPGMA).

## 4. Conclusions

This study compared *H. arenarium* extracts obtained from inflorescences, mother plant shoots, in vitro-derived plantlets, and callus cultures. Phytochemical profiling by UHPLC-DAD-ESI-MS^3^, together with biological assays, showed clear tissue- and line-dependent differences in both metabolite composition and biological activity. Ex vitro plant material, especially inflorescences and mother plant shoots, had the richest flavonoid and chalcone profile and showed the strongest activity in most of the tested assays. These results confirm the importance of differentiated plant organs as sources of phenolic compounds with potential skin-related activity.

The in vitro-derived biomass did not reproduce the full chemical profile of the traditionally used inflorescences. Characteristic flower-associated compounds, including isosalipurposide and naringenin-derived flavanones, were detected only in ex vitro plant material. Free apigenin was quantified only in the inflorescence extract. However, callus cultures retained the ability to synthesize selected phenolic metabolites. The Ha-C-1 callus line combined the highest biomass increase with the highest accumulation of chlorogenic acid and isochlorogenic acid A. This indicates that selected callus cultures may be useful as a controlled source of caffeoylquinic acid derivatives.

The biological assays confirmed that extract activity depended strongly on the type of plant material and extract concentration. The extracts did not markedly reduce HaCaT cell viability up to 500 µg/mL. Selected samples inhibited collagenase and hyaluronidase activity, modulated cytokine secretion, and affected keratinocyte migration in the scratch assay. The strongest collagenase inhibition was observed for Ha-I and Ha-MP, whereas hyaluronidase inhibition was most pronounced for Ha-MP, followed by selected in vitro-derived extracts and Ha-I. The cytokine response was selective. Ha-I reduced both IL-6 and IL-8 secretion at higher concentrations, whereas Ha-P-12 and Ha-C-12 were particularly effective in lowering IL-8 secretion. In the scratch assay, selected extracts enhanced wound closure, while Ha-C-12 inhibited wound closure at the lowest tested concentrations. This confirms the need for line-specific biological evaluation.

Nevertheless, *H. arenarium* callus cultures cannot be regarded as direct substitutes for the traditionally used flower material. However, they may serve as a starting material for the production of selected phenolic acids and for further optimization of bioactive biomass. Further work should focus on line selection, elicitation, light conditions, plant growth regulator composition, and possible scale-up to suspension cultures. These steps may increase the accumulation of target metabolites and help develop standardized *H. arenarium* extracts while reducing the need to collect plant material from natural populations.

## Figures and Tables

**Table 1:** Compounds confirmed or tentatively identified in *H. arenarium* extracts using UHPLC-DAD-ESI-MS^3^ in negative ion mode. The presence of each compound is indicated in individual extracts from: inflorescences (Ha-I), maternal plants (Ha-MP), plantlet lines (Ha-P-1; Ha-P-4; Ha-P-12) and callus tissue lines (Ha-C-1; Ha-C-4; Ha-C-12). ‘+’ indicates detection.

No	Compound	t_R_ [min]	UV–Vis [nm]	[M−H]^−^ *m*/*z*	MS^2^ Ions *m*/*z*	MS^3^ Ions *m*/*z*	Ha-I	Ha-MP	Ha-P-1	Ha-P-4	Ha-P-12	Ha-C-1	Ha-C-4	Ha-C-12	Ref.
**1**	5-*O*-(4′-*O*-Glucosylhydroxydihydrocaffeoyl)quinic acid	1.4	255	533	371, 341, 191, 173, 155			+	+	+	+				[17]
**2**	Quinic acid	1.4	255	191	173, 155		+	+							[18]
**3**	Saccharose	1.6	-	341	215, 197, 179, 161		+	+	+	+	+	+	+	+	[19]
**4**	Raffinose (Melitose)	1.7	-	503	341, 323, 221, 215, 179, 161							+	+	+	[19]
**5**	Isomaltotriose	1.9	-	503	387, 341, 323, 221, 179, 161							+	+	+	[20]
**6**	Unknown	2.4	265	290	272, 254, 230, 200, 128		+	+	+	+		+	+	+	
**7**	Unknown	8.1	251	263	207, 161, 113, 101		+								
**8**	Unknown	12.5	257, 285sh	327	207, 165, 121		+								
**9**	Unknown	17.7	280	359	299, 239, 199				+	+	+	+	+		
**10**	Unknown	18.7	-	445	427, 385, **269**, 175	161							+	+	
**11**	Unknown	19.0	-	445	359, 323, 264		+								
**12**	Unknown	19.9	257, 280sh	341	207, 179		+								
**13**	Unknown	20.7	335	455	423, 311, 293, 163				+	+	+				
**14**	Chlorogenic acid (3-*O*-Caffeoylquinic acid) *	21.6	300sh, 325	353	**191**, 179	173	+	+	+	+	+	+	+	+	[18]
**15**	Apigenin 7-*O*-xyloside	23.7	300sh, 325	401	269, 233, 161		+	+	+	+	+	+		+	[17]
**16**	Everlastoside E	24.9	330	431	385, 369, 329, 243, 203, 125		+	+	+	+	+				[21]
**17**	Unknown	25.8	335	569	551, 371, 327, 197, 173			+	+	+					
**18**	Unknown	26.3	333	401	179			+							
**19**	Unknown	26.8	-	517	**385**, 293, 223, 205	223, 205		+	+	+	+				
**20**	Unknown	28.3	264, 300sh	537	375, 327		+						+	+	
**21**	Everlastoside D	30.2	277	393	311, 149			+		+	+				[21]
**22**	3-*O*-Feruloylquinic acid	30.4	298sh, 325	367	193, **191,** 173	127						+	+		[17]
**23**	1,4-Di-*O*-caffeoylquinic acid	31.0	300sh, 325	515	**353**, 191, 179	191, 179		+	+	+	+				[17]
**24**	Unknown	31.8	300sh, 370	517	423, 293, 287, **261**, 149	217	+	+	+	+	+				
**25**	Unknown	33.2	273	395	269, **233**	203, 185				+	+	+	+	+	
**26**	Unknown	33.6	312	481	317, 299, 281, 179		+								
**27**	Everlastoside A	35.5	336	401	383, 357, 261, 221, 195, 177			+							[21]
**28**	Helichrysin A ((+)-Naringenin 5-*O*-β-D-glucoside)	35.9	281	433	271	151	+	+							[2,18]
**29**	Quercetin 3-*O*-β-D-glucuronide 6″-*n*-butyl ester	36.7	275, 340	533	**463**	301		+							[22]
**30**	Helichrysin B (salipurposide, (−)-Naringenin 5-*O*-β-D-glucoside)	37.7	280	433	313, **271**, 151	151, 119	+	+							[18,23]
**31**	Unknown	38.6	275, 300sh	565	505, 339, 327, 207							+	+	+	
**32**	Unknown	39.9	259, 295	395	245				+	+	+				
**33**	Unknown	40.5	340	511	451, **379**, 217	259, 241, 217, 201				+					
**34**	Naringenin 4′-*O*-β-D-glucoside	40.7	293	433	**271**	150	+								[23,24]
**35**	Isoquercitrin (Quercetin 3-*O*-β-D-glucoside)	40.8	339	463	**301**	271, 255, 179, 151	+	+							[23]
**36**	Unknown	42.0	264	613	553					+		+	+		
**37**	Unknown	42.5	332	519	**357**	311, 151								+	
**38**	Unknown	42.6	349	537	293, 243				+	+	+				
**39**	Prunin (Naringenin 7-*O*-β-D-glucoside)	43.9		433	**271**	177, 151	+								[18,23]
**40**	Astragalin (Kaempferol 3-*O*-glucopyranoside)	45.2	264, 344	447	255, 285, 327		+	+							[18]
**41**	Isochlorogenic acid B (3,4-Di-*O*-caffeoylquinic acid)	45.6	300sh, 325	515	**353**	191, 179, 173	+	+	+	+	+	+	+	+	[18]
**42**	Apigenin 7-*O*-glucoside	45.9	330	431	**269**	197	+								[18]
**43**	Apigenin 7-*O*-glucuronide	47.9	335	445	**269**, 175	197, 149, 117	+								[25]
**44**	3,5-Di-*O*-caffeoyl-4-malonylquinic acid	48.5	330	601	557, 515, **439**, 395, 233, 191, 179	395, 233		+	+	+	+				[25]
**45**	Isochlorogenic acid A (3,5-Di-*O*-caffeoylquinic acid) *	49.1	300sh, 325	515	**353**	191, 179, 173	+	+	+		+				[17]
**46**	Unknown	49.4	327	501	339, 323, 221, 177	333,7; 176,5		+				+	+	+	
**47**	Isochlorogenic acid C (4,5-Di-*O*-caffeoylquinic acid)	49.7	300sh, 325	515	**353**	191, 179, 173	+	+	+	+	+	+	+	+	[17]
**48**	Isosalipurposide (Chalconaringenin 2′-*O*-glucoside)	51.6	368	433	313, **271**	177, 165, 151, 107	+	+							[18]
**49**	Unknown	54.0	275, 325sh	479	419, 299, 287, 269, 257							+	+		
**50**	Kaempferol 3-*O*-gentiobioside	56.3	335	609	**447**, 323, 285	327, 285, 255	+								[5]
**51**	Unknown	60.5	-	551	431, 335, 213				+	+	+				
**52**	Unknown	61.4	-	551	431, 335, 213							+	+	+	
**53**	Kaempferol 7-*O*-rutinoside	61.7	293	593	**447**, 285	327, 285, 255	+								[26]
**54**	Naringenin	61.7	293	271	177, 151, 107		+								[24]
**55**	Tiliroside (Kaempferol 3-*O*-(6″-*O*-*p*-coumaroyl)-β-D-glucoside)	63.0	325	593	**447**, 285	327, 285, 255	+								[18]
**56**	Apigenin *	64.8	266, 336	269	225, 159		+	+							[18]
**57**	7-*O*-Glucoxy-5-hydroxy-1(3H)-isobenzofuranone	66.2	328	327	291, 229, 211, 171			+	+	+				+	[18,27]
**58**	Isorhamnetin 3-*O*-glucoside-7-*O*-xyloside	67.5	327	609	477, 447, 315				+	+					[18]
**59**	Rhamnetin 3-*O*-(6″-*O*-xylosyl)glucoside	67.9	327	609	315				+	+	+				[18]
**60**	Quercetin 5,7-dimethyl ether	68.4	290, 310sh	329	311, 293, 229, 211		+	+	+	+	+	+	+	+	[28]
**61**	Prunin 6″-*O*-gallate (Naringenin 7-*O*-β-D-(6″-*O*-galloyl)glucoside)	72.5	290, 325	585	445, **433**, 313, 271	313, 271	+								[22]
**62**	Unknown	72.9	275, 325	293	235			+				+	+	+	
**63**	Prunin derivative	74.2	285, 370	599	433, 313, 271		+								
**64**	Unknown	75.8	-	487	469, 427								+		
**65**	Rhamnetin	77.7	275, 325, 375sh	315	297, 279, 171				+	+	+				[18]
**66**	Unknown	78.6	-	293	275, 231, 211, 171			+		+		+	+	+	
**67**	Unknown	79.0	278	513	277, 253				+	+	+				
**68**	Unknown	79.8	-	309	**291**	273, 247, 219						+	+	+	
**69**	Helispiroketal G	80.1	-	453	255							+	+	+	[29]
**70**	Unknown	80.2	-	421	255		+	+							
**71**	6-Hydroxy-7-methoxy-2-(2-phenylethyl)chromone	80.8	288	295	277, 171		+	+	+	+	+	+	+	+	[18]
**72**	3-[2,4-Dihydroxy-6-methoxy-3-(3-methylbut-2-enyl)-5-(2-methylbutyryl)benzyl]-6-ethyl-4-methoxy-5-methylpyran-2-one	81.2	285	471	409, 191			+	+	+	+	+	+	+	[30]
**73**	Taxifolin (Dihydroquercetin)	81.2	287	303	259, 179		+	+							
**74**	Fargesone A	81.2	287	371	303, 259		+								[18]
**75**	Unknown	81.5	-	425	407, **319**	277, 179			+	+	+				
**76**	Unknown	81.8	277	293	275, 249							+	+	+	
**77**	Arenol A	84.0	290	387	247, 235		+		+		+				[31]
**78**	Homoarenol	85.7	292	401	247		+								[31]
**79**	Fargesone B	85.9	292	371	302, 259		+		+		+				[18]
**80**	Arzanol	86.8	290, 230	401	255		+	+	+	+					[28]
**81**	Petiolactone A	87.0	-	449	**297**	227, 219, 145	+	+	+	+	+				[32]
**82**	Unknown	87.6	-	277	233							+	+	+	
**83**	Unknown	87.8	283	433	364, 321, 253		+		+		+				
**84**	22,22-Dimethylitalipyron	87.9	283	427	281, 194		+		+	+	+				[33]
**85**	Obtusifolin trimethyl ether	88.7	290, 325sh	463	297		+								[32]
**86**	Methylarzanol	89.4	-	415	276, 233				+	+	+				[28]
**87**	Arenol C	89.6	285	429	289, **277**	233, 207	+				+				[34]
**88**	Helispiroketal C	90	295	399	330, 287		+		+		+				[29]
**89**	Unknown	90	295	279	261			+		+					
**90**	Unknown	90	-	495	473						+				

* comparison with chemical standard have been made; “-” spectrum unclear or overlapped with signals from co-eluting compounds; sh—shoulder in UV–Vis spectrum. Bolded MS^2^ ions indicate precursor ions selected for MS^3^ fragmentation.

## Data Availability

The dataset is available on reasonable request from the authors.

## Supplementary Materials

The following supporting information can be downloaded at: https://www.mdpi.com/article/10.3390/molecules31152625/s1, **Figure S1.** Negative-ion MS^2^ spectrum of compound **C_48_**, tentatively assigned as isosalipurposide (chalconaringenin 2′-*O*-glucoside), with the proposed structures of the major product ions. The blue diamond indicates the deprotonated precursor ion [M−H]^−^, whereas the red diamond indicates the principal diagnostic product ion. **Figure S2.** Negative-ion MS^2^ and MS^3^ spectra of compound **C_61_**, tentatively identified as prunin 6″-*O*-gallate [naringenin 7-*O*-β-D-(6″-*O*-galloyl)glucoside], together with the proposed fragmentation pathway and structures of selected product ions. **Figure S3.** Chemical structures and negative-ion MS^2^ spectra of compounds **C_42_** and **C_43_**, tentatively identified as apigenin 7-*O*-glucoside and apigenin 7-*O*-glucuronide, respectively. The characteristic neutral losses of glucose and glucuronic acid residues are indicated. **Figure S4.** Negative-ion MS^2^ and MS^3^ spectra of compound **C_35_**, tentatively identified as isoquercitrin (quercetin 3-*O*-β-D-glucoside), with assignments of selected aglycone-derived product ions and proposed fragmentation sites. **Figure S5.** Chemical structures and negative-ion MS^2^ and MS^3^ spectra of compounds **C_53_** and **C_55_**, tentatively identified as kaempferol 7-*O*-rutinoside and tiliroside, respectively. **Figure S6.** Negative-ion MS^2^ and MS^3^ spectra of compound **C_44_**, tentatively identified as 3,5-di-*O*-caffeoyl-4-*O*-malonylquinic acid, together with the proposed fragmentation pathway and structures of the selected most abundant product ions. **Figure S7.** Negative-ion MS^2^ and MS^3^ spectra of compounds **C_77_**, **C_78_**, and **C_87_**, tentatively identified as arenol A, homorenol, and arenol C, respectively, with proposed structures of the selected most abundant product ions. **Figure S8.** Negative-ion MS^2^ spectrum of compound **C_57_**, tentatively identified as 7-*O*-glucoxy-5-hydroxy-1(3H)-isobenzofuranone, with proposed structures of selected product ions. **Figure S9.** Negative-ion MS^2^ spectra of compounds **C_16_** and **C_21_**, tentatively identified as everlastoside E and everlastoside D, respectively, together with the proposed fragmentation pathways and structures of selected product ions. **Figure S10.** Negative-ion MS^2^ spectrum of compound **C_27_**, tentatively identified as everlastoside A, with proposed structures of the selected most abundant product ions. **Figure S11.** Chemical structures and negative-ion MS^2^ spectra of compounds **C_69_** and **C_88_**, tentatively identified as helispiroketal G and helispiroketal C, respectively. **Figure S12.** Negative-ion MS^2^ and MS^3^ spectra of compound **C_81_**, tentatively identified as petiolactone A, with the proposed structure of a selected product ion, and negative-ion MS^2^ spectra of compounds **C_74_** and **C_79_**, tentatively identified as fargesone A and fargesone B, respectively. **Table S1.** Linearity, LOD, and LOQ data for the used standards.

## Abbreviations

The following abbreviations are used in this manuscript:

**Abbreviation**	**Definition**
2,4-D	2,4-dichlorophenoxyacetic acid
ACN	acetonitrile
ANOVA	analysis of variance
BAP	6-benzylaminopurine
BPC	base peak chromatogram
ChC	collagenase from Clostridium histolyticum
CPT	camptothecin
DAD	diode-array detector/detection
DE	dry extract
DMABA	p-dimethylaminobenzaldehyde
DMEM	Dulbecco’s modified Eagle medium
DMSO	dimethyl sulfoxide
DPBS	Dulbecco’s phosphate-buffered saline
DPPH	2,2-diphenyl-1-picrylhydrazyl radical
DW	dry weight
EGCG	epigallocatechin gallate
ELISA	enzyme-linked immunosorbent assay
ESI	electrospray ionization
FALGPA	N-[3-(2-furyl)acryloyl]-Leu-Gly-Pro-Ala
FBS	fetal bovine serum
FRAP	ferric reducing antioxidant power
FW	fresh weight
GAE	gallic acid equivalents
Ha-C-1, Ha-C-4, Ha-C-12	*H. arenarium* callus culture lines 1, 4 and 12
Ha-I	extract obtained from *H. arenarium* inflorescences
Ha-MP	extract obtained from shoots of the *H. arenarium* mother plant
Ha-P-1, Ha-P-4, Ha-P-12	*H. arenarium* in vitro-derived plantlet lines 1, 4 and 12
HaCaT	spontaneously immortalized human epidermal keratinocytes
HC	hydrocortisone
HPLC-MS/MS	high-performance liquid chromatography coupled with tandem mass spectrometry
IFN-γ	interferon gamma
IL-6	interleukin 6
IL-8	interleukin 8
IPA	isopropanol
LC-MS	liquid chromatography-mass spectrometry
LOD	limit of detection
LOQ	limit of quantification
MeOH	methanol
MS medium	Murashige and Skoog medium
MS^2^/MS^3^	tandem/multistage mass spectrometry
MTT	thiazolyl blue tetrazolium bromide
NaClO	sodium hypochlorite
NST	non-stimulated control
NT	non-treated control
OD	optical density
PVDF	polyvinylidene fluoride
QE	quercetin equivalents
R^2^	correlation coefficient for the linear model
SD	standard deviation
ST	stimulated control
TE	Trolox equivalents
TFC	total flavonoid content
TPC	total phenolic content
TNF-α	tumor necrosis factor alpha
UHPLC-DAD-ESI-MS^3^	ultra-high-performance liquid chromatography coupled with diode-array detection and electrospray ionization multistage mass spectrometry
UV–Vis	ultraviolet–visible
