# Supplementary material for: Antioxidant and Anti-Aging Activity of Helichrysum arenarium (L.) Moench: Comparative Evaluation of Extracts from Shoots, Inflorescences, Plantlets and Callus Tissue
Source: Molecules. 2026 Jul 28;31(15):2625. doi: 10.3390/molecules31152625 (PMC13468370; doi:10.3390/molecules31152625)
Supplement: Supplementary file 1 [file molecules-31-02625-s001.zip › molecules-4445076-supplementary.pdf]

Antioxidant and Anti-Aging Activity of *Helichrysum arenarium* (L.) Moench: Comparative Evaluation of Extracts from Shoots, Inflorescences, Plantlets and Callus Tissue

Joanna Jabłońska [1,†], Maciej Obrębski [1,2,†], Rafał M. Kiełkiewicz [1,*], Irena Macias [1], Julia Bogusz [3], Weronika Skowrońska [1] and Katarzyna Sykłowska-Baranek [1]

[1] Department of Pharmaceutical Biology, Faculty of Pharmacy, Medical University of Warsaw, 1 Banacha St., 02-097 Warsaw, Poland; s082470@student.wum.edu.pl (J.J.); maciej.obrebski@wum.edu.pl (M.O.); s091804@student.wum.edu.pl (I.M.); weronika.skowronska@wum.edu.pl (W.S.); katarzyna.syklowska-baranek@wum.edu.pl (K.S.-B.)
[2] MicrobiotaLab, Department of Pharmaceutical Microbiology and Bioanalysis, Faculty of Pharmacy, Medical University of Warsaw, 1 Banacha St., 02-097 Warsaw, Poland
[3] Faculty of Biochemistry, Biophysics and Biotechnology at the Jagiellonian University, 7 Gronostajowa St., 30-387 Cracow, Poland; julia.bogusz@student.uj.edu.pl
* Correspondence: rafal.kielkiewicz@wum.edu.pl
† These authors contributed equally to this work.

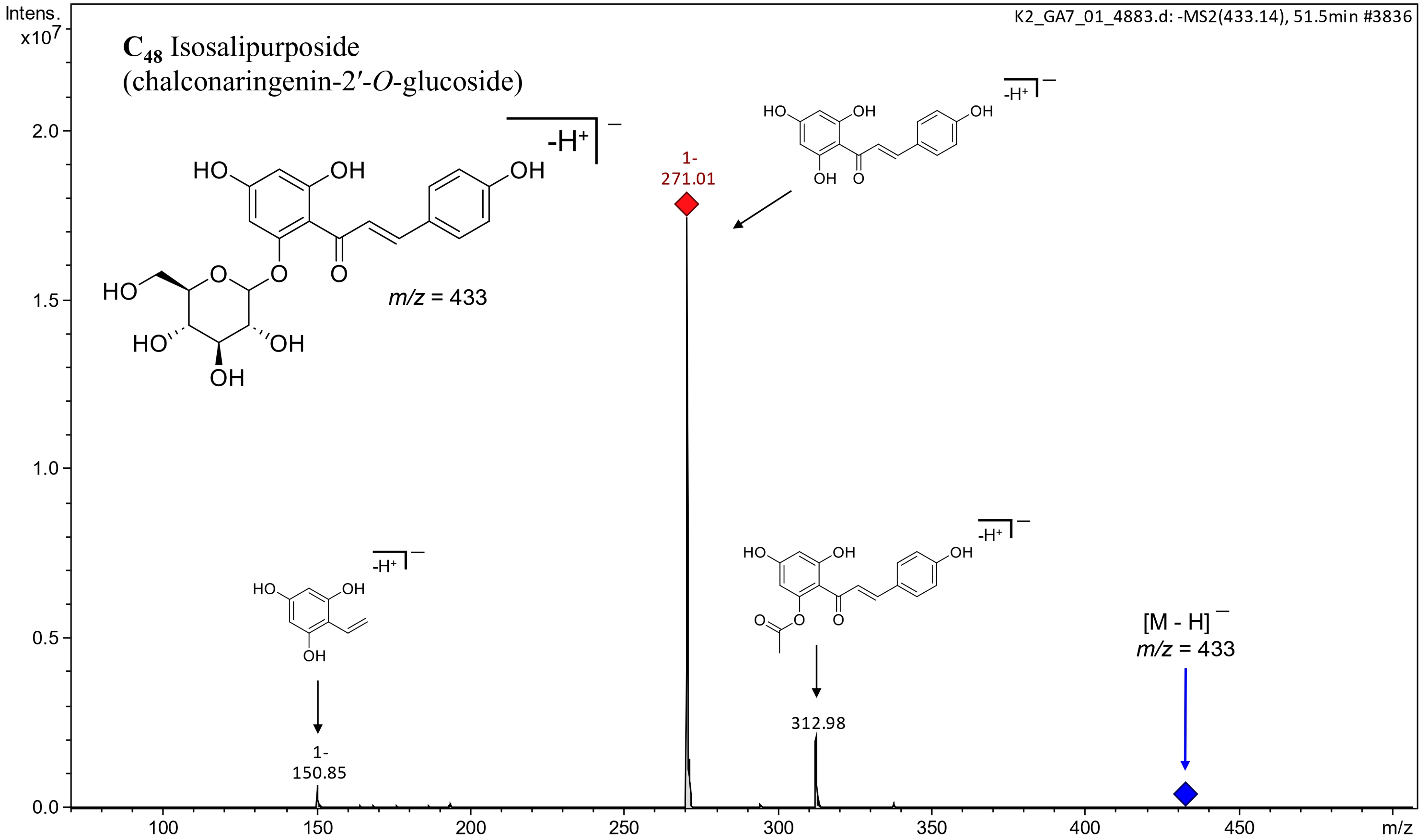

Figure S1. Negative-ion MS² spectrum of compound C48, tentatively assigned as isosalipurposide (chalconaringenin 2′-*O*-glucoside), with the proposed structures of the major product ions. The blue diamond indicates the deprotonated precursor ion [M − H]⁻, whereas the red diamond indicates the principal diagnostic product ion.

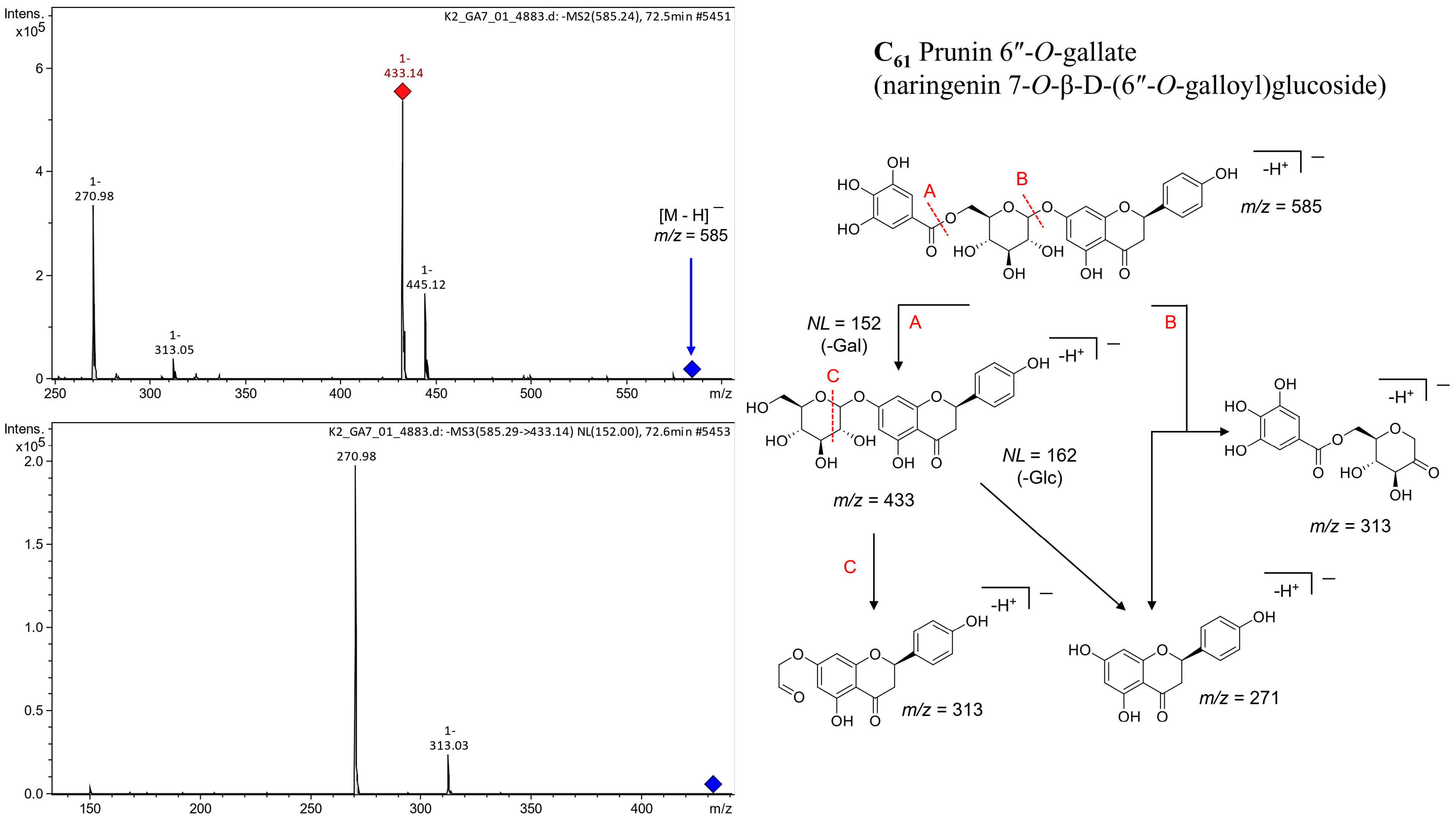

Figure S2. Negative-ion MS² and MS³ spectra of compound C_{61}, tentatively identified as prunin 6″-*O*-gallate [naringenin 7-*O*-β-D-(6″-*O*-galloyl)glucoside], together with the proposed fragmentation pathway and structures of selected product ions.

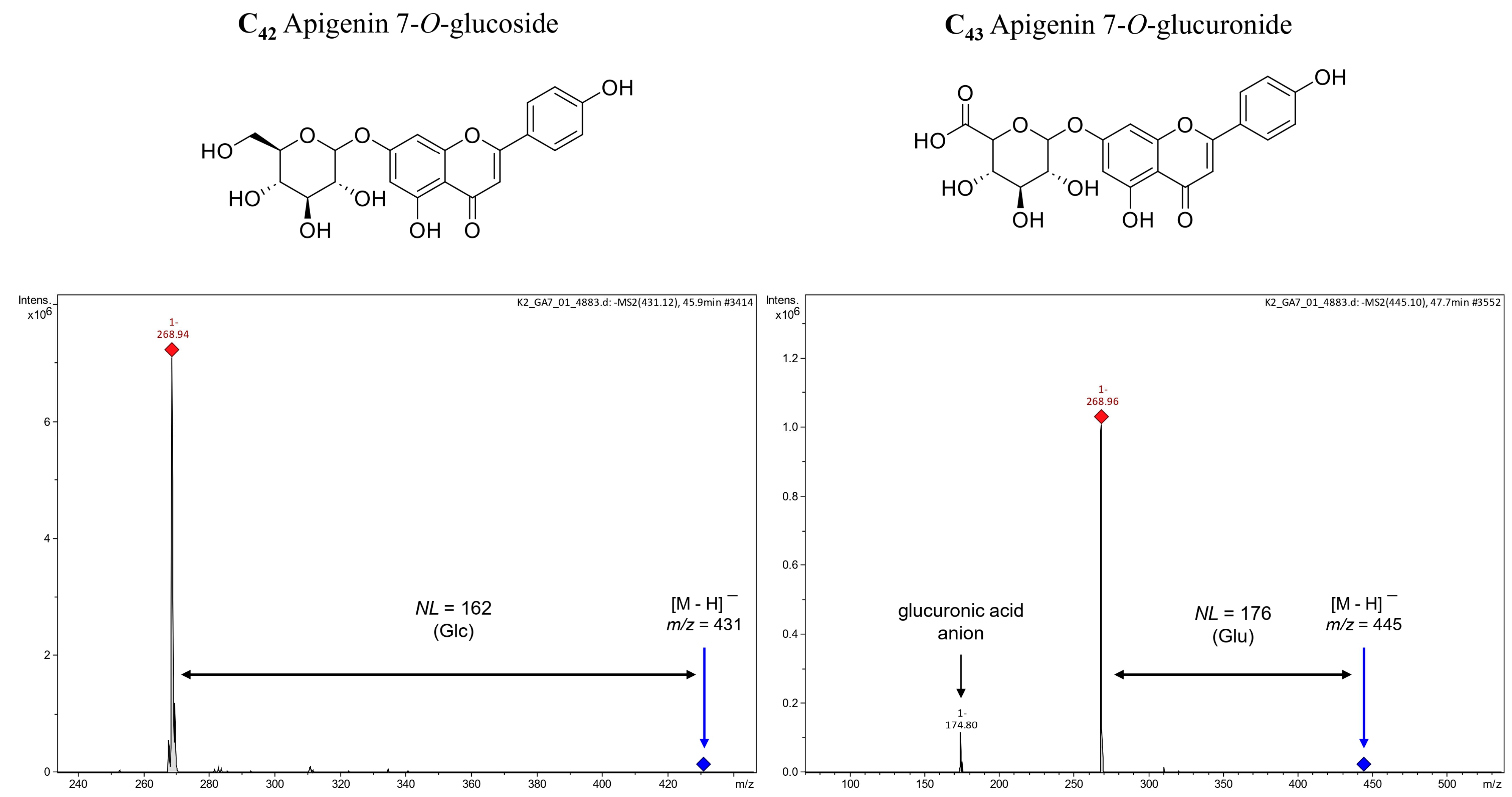

Figure S3. Chemical structures and negative-ion MS² spectra of compounds C42 and C43, tentatively identified as apigenin 7-*O*-glucoside and apigenin 7-*O*-glucuronide, respectively. The characteristic neutral losses of glucose and glucuronic acid residues are indicated.

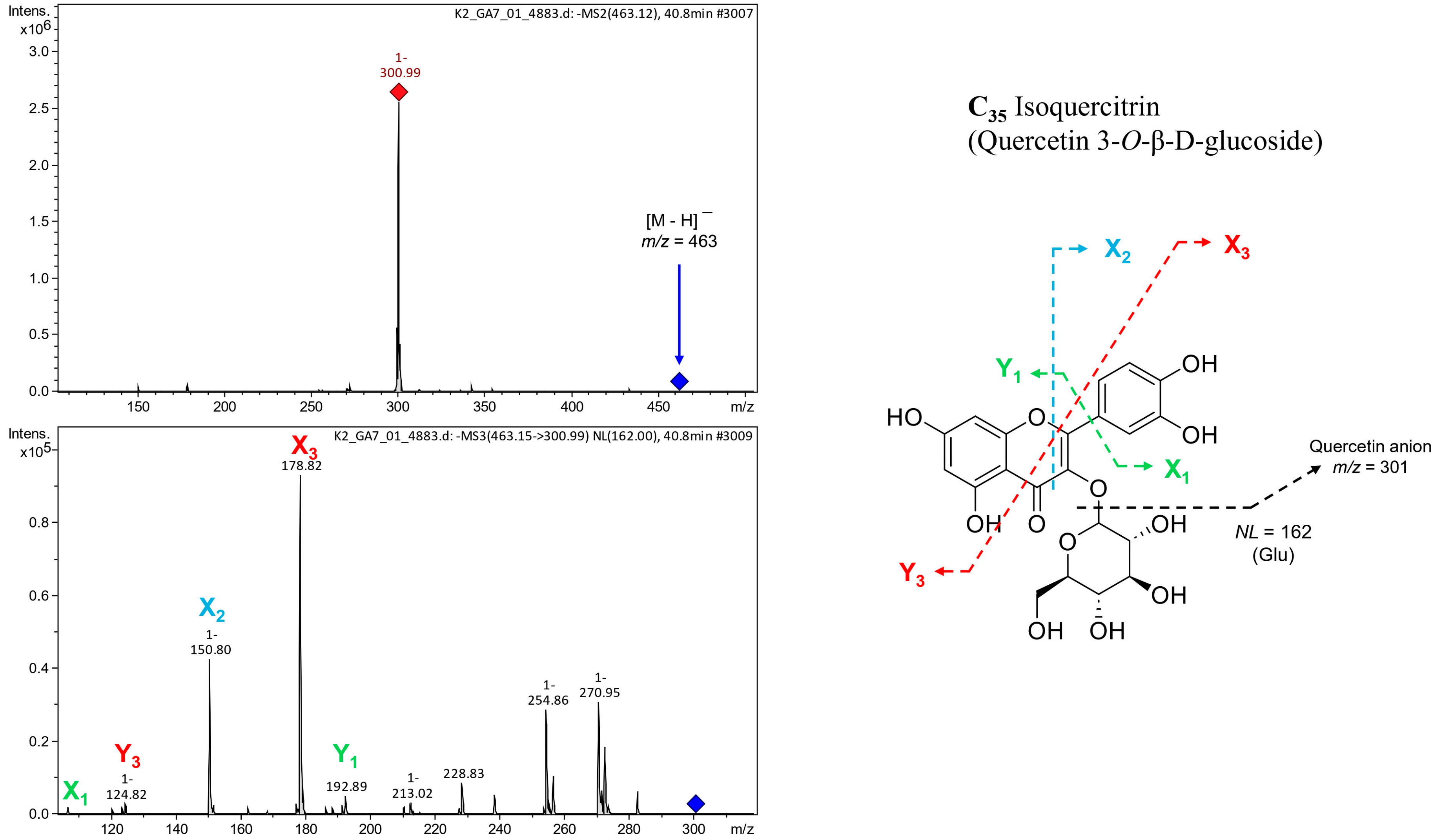

Figure S4. Negative-ion MS² and MS³ spectra of compound C35, tentatively identified as isoquercitrin (quercetin 3-*O*-β-D-glucoside), with assignments of selected aglycone-derived product ions and proposed fragmentation sites.

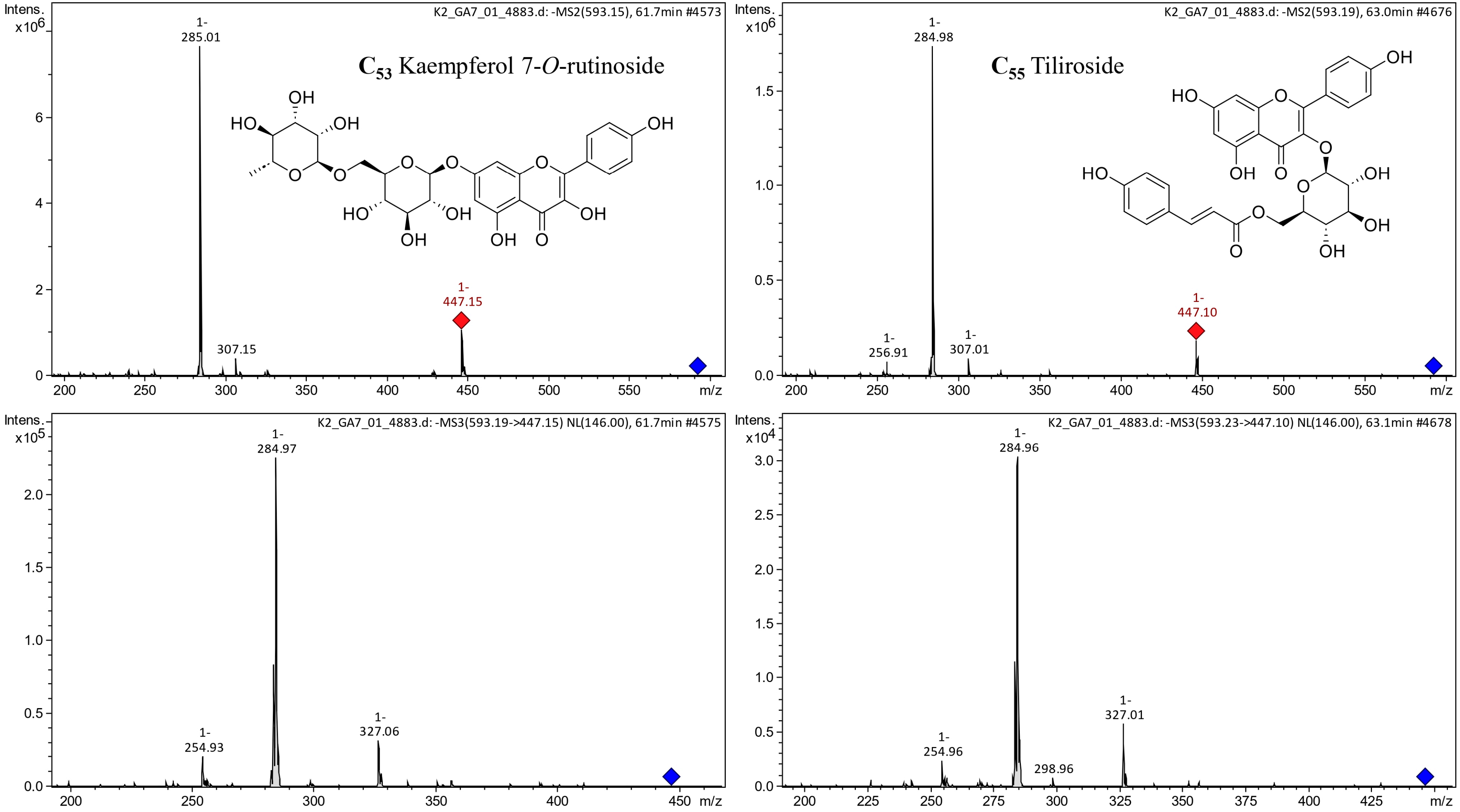

Figure S5. Chemical structures and negative-ion MS² and MS³ spectra of compounds C_{53} and C_{55}, tentatively identified as kaempferol 7-*O*-rutinoside and tiliroside, respectively.

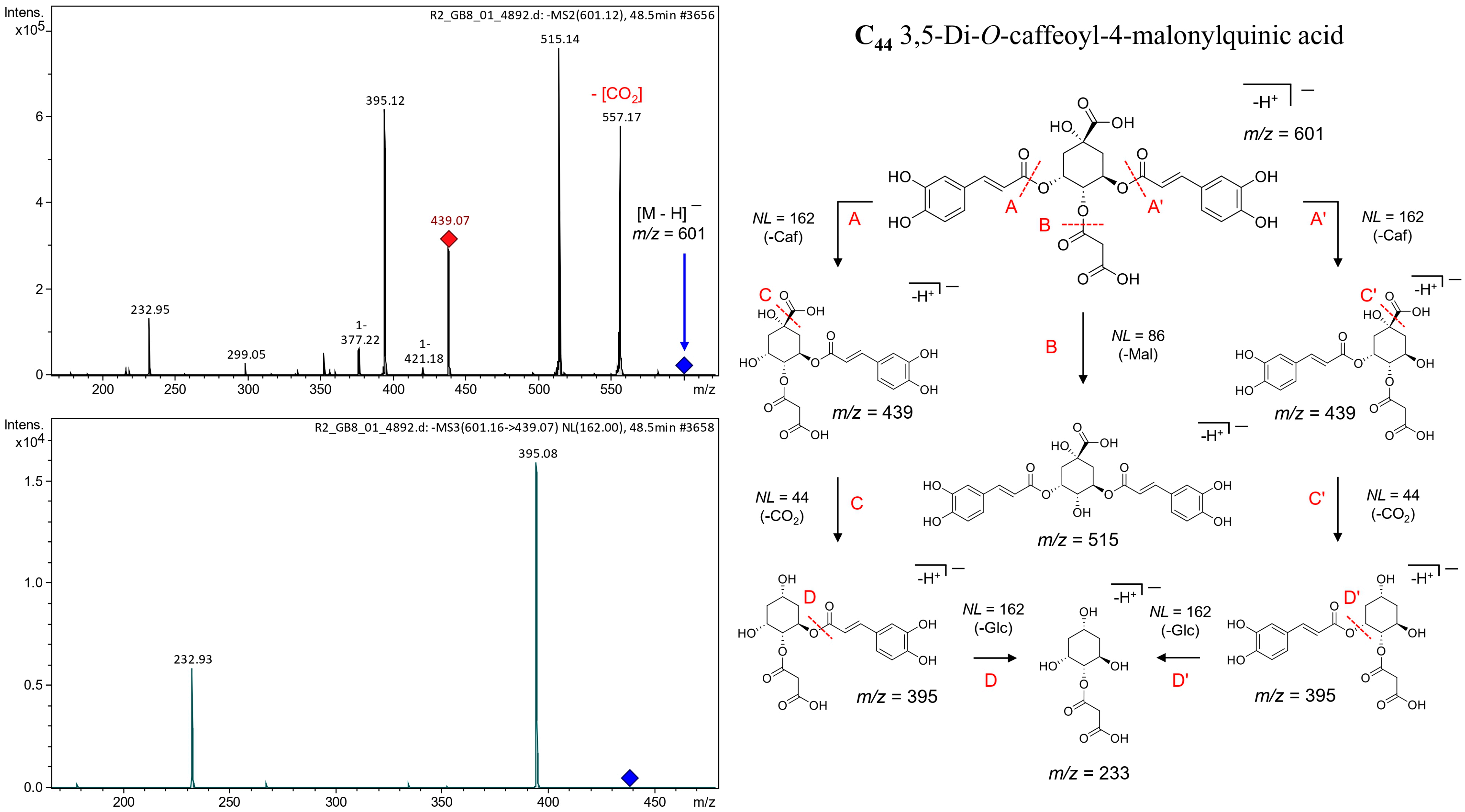

Figure S6. Negative-ion MS² and MS³ spectra of compound C44, tentatively identified as 3,5-di-*O*-caffeoyl-4-*O*-malonylquinic acid, together with the proposed fragmentation pathway and structures of the selected most abundant product ions.

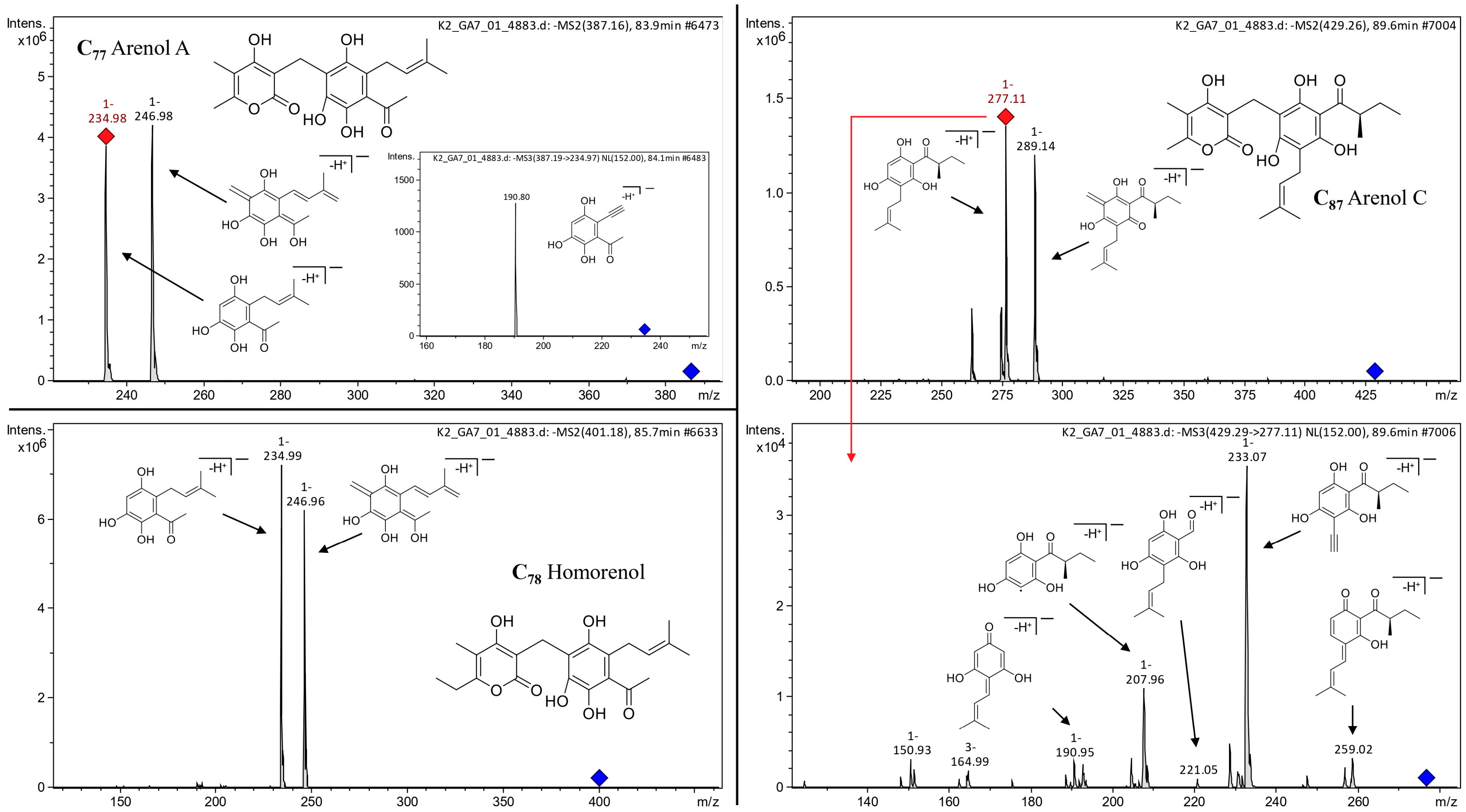

Figure S7. Negative-ion MS² and MS³ spectra of compounds C_{77}, C_{78}, and C_{87}, tentatively identified as arenol A, homorenol, and arenol C, respectively, with proposed structures of the selected most abundant product ions.

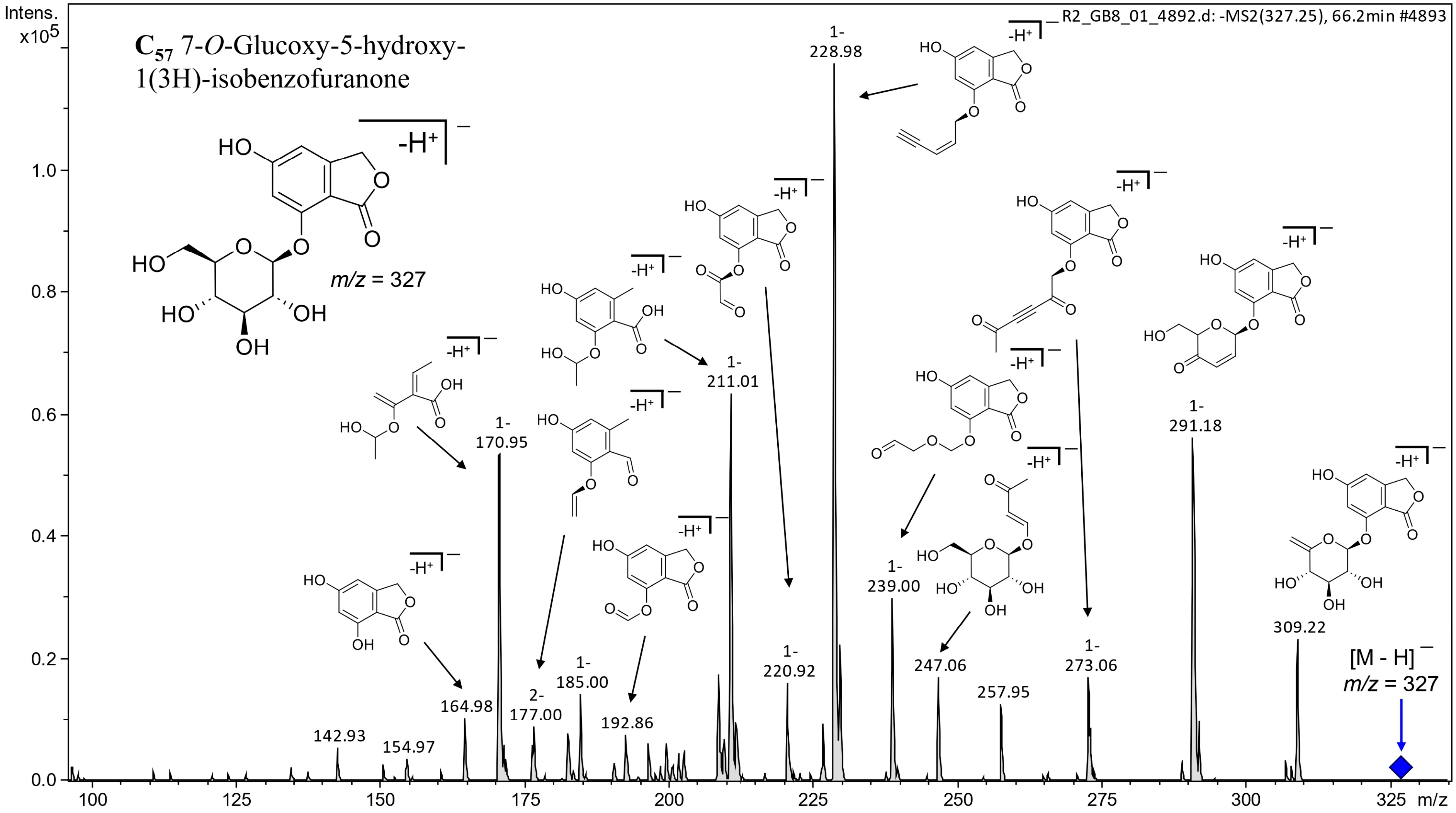

Figure S8. Negative-ion MS² spectrum of compound C_{57}, tentatively identified as 7-*O*-glucoxy-5-hydroxy-1(3H)-isobenzofuranone, with proposed structures of selected product ions.

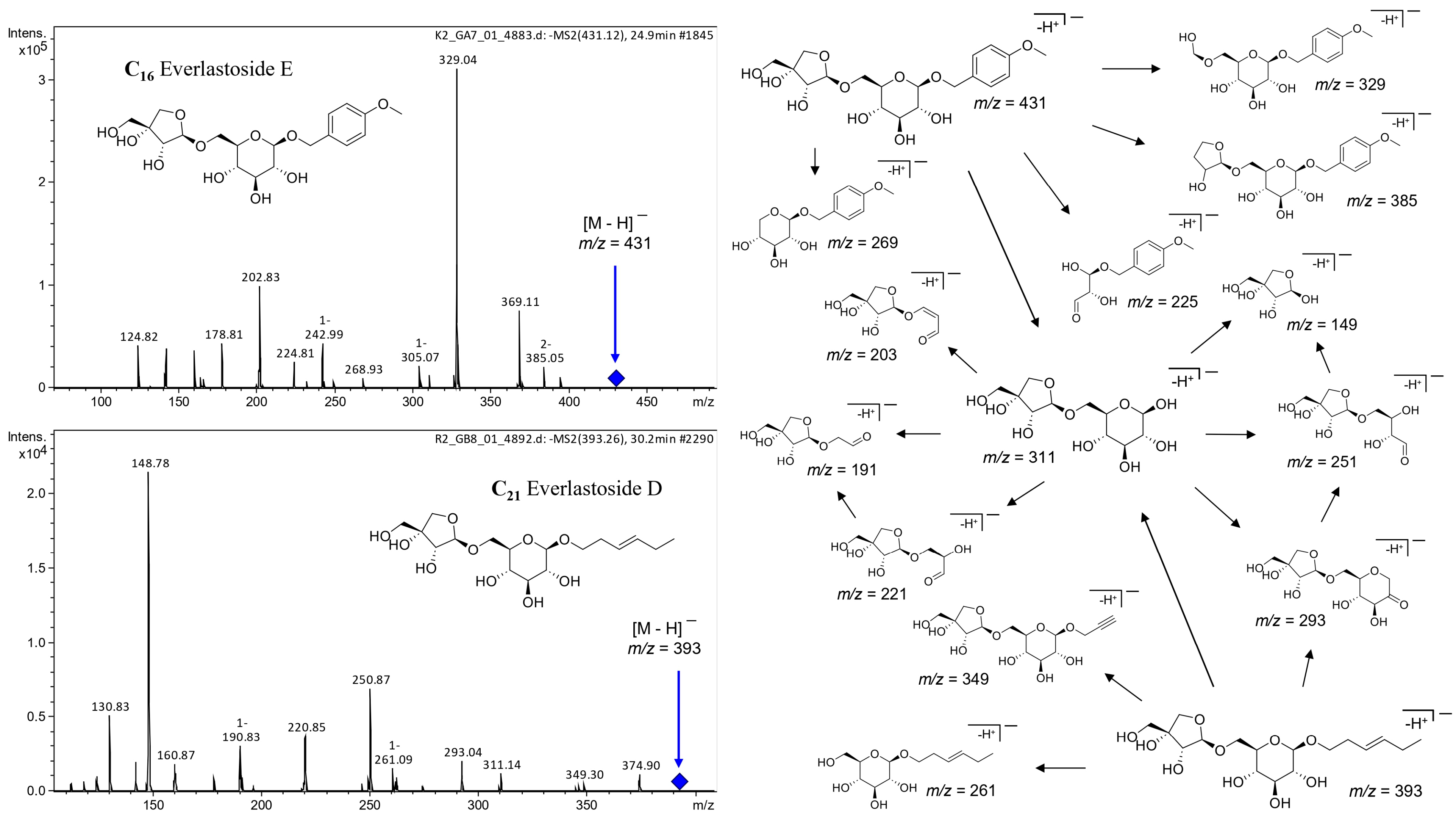

Figure S9. Negative-ion MS^2 spectra of compounds C_{16} and C_{21}, tentatively identified as everlastoside E and everlastoside D, respectively, together with the proposed fragmentation pathways and structures of selected product ions.

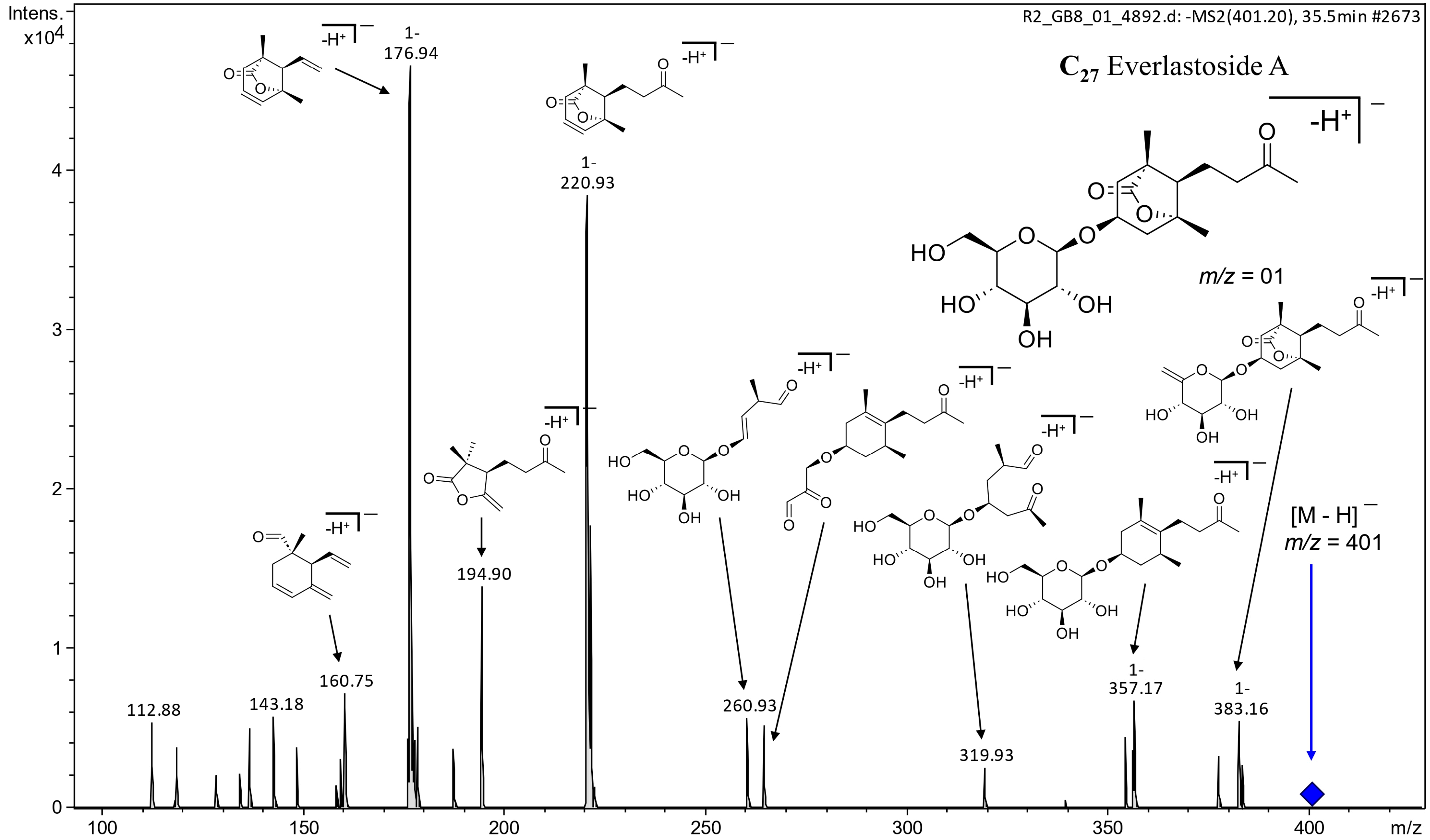

Figure S10. Negative-ion MS² spectrum of compound C_{27}, tentatively identified as everlastoside A, with proposed structures of the selected most abundant product ions.

C_{69} Helispiroketal G

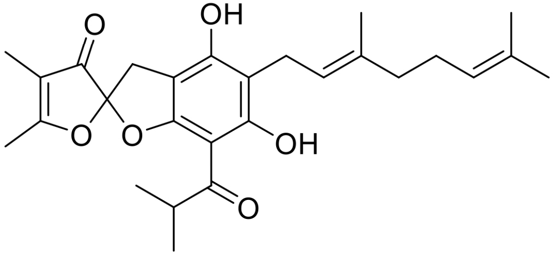

C_{88} Helispiroketal C

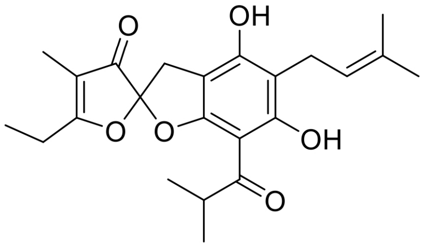

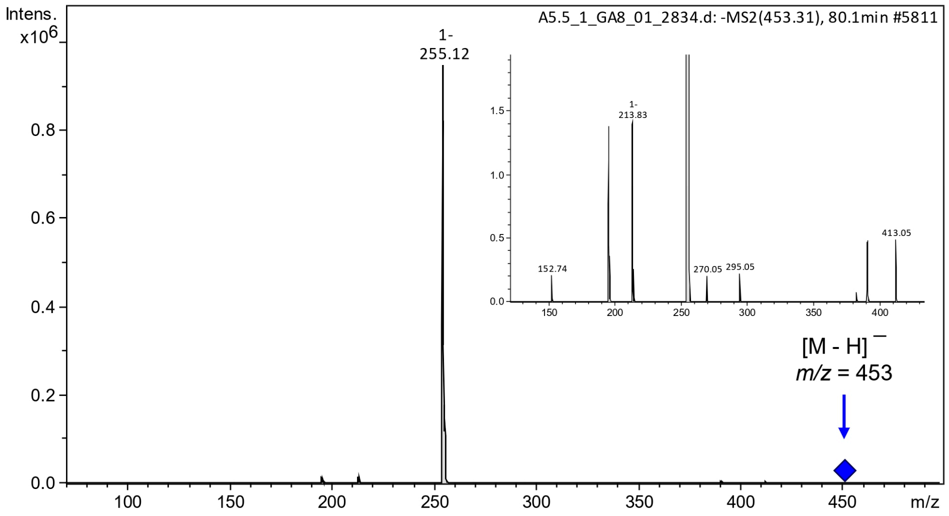

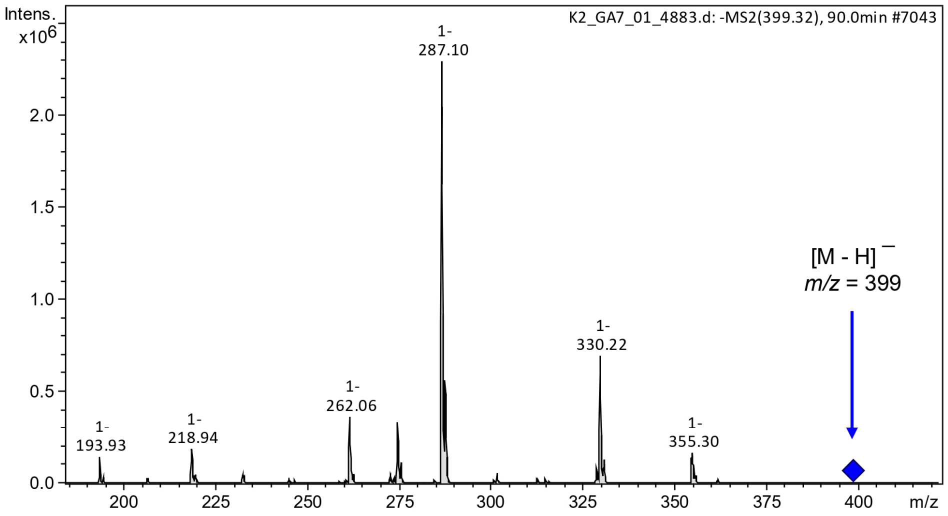

Figure S11. Chemical structures and negative-ion MS² spectra of compounds C_{69} and C_{88}, tentatively identified as helispiroketal G and helispiroketal C, respectively.

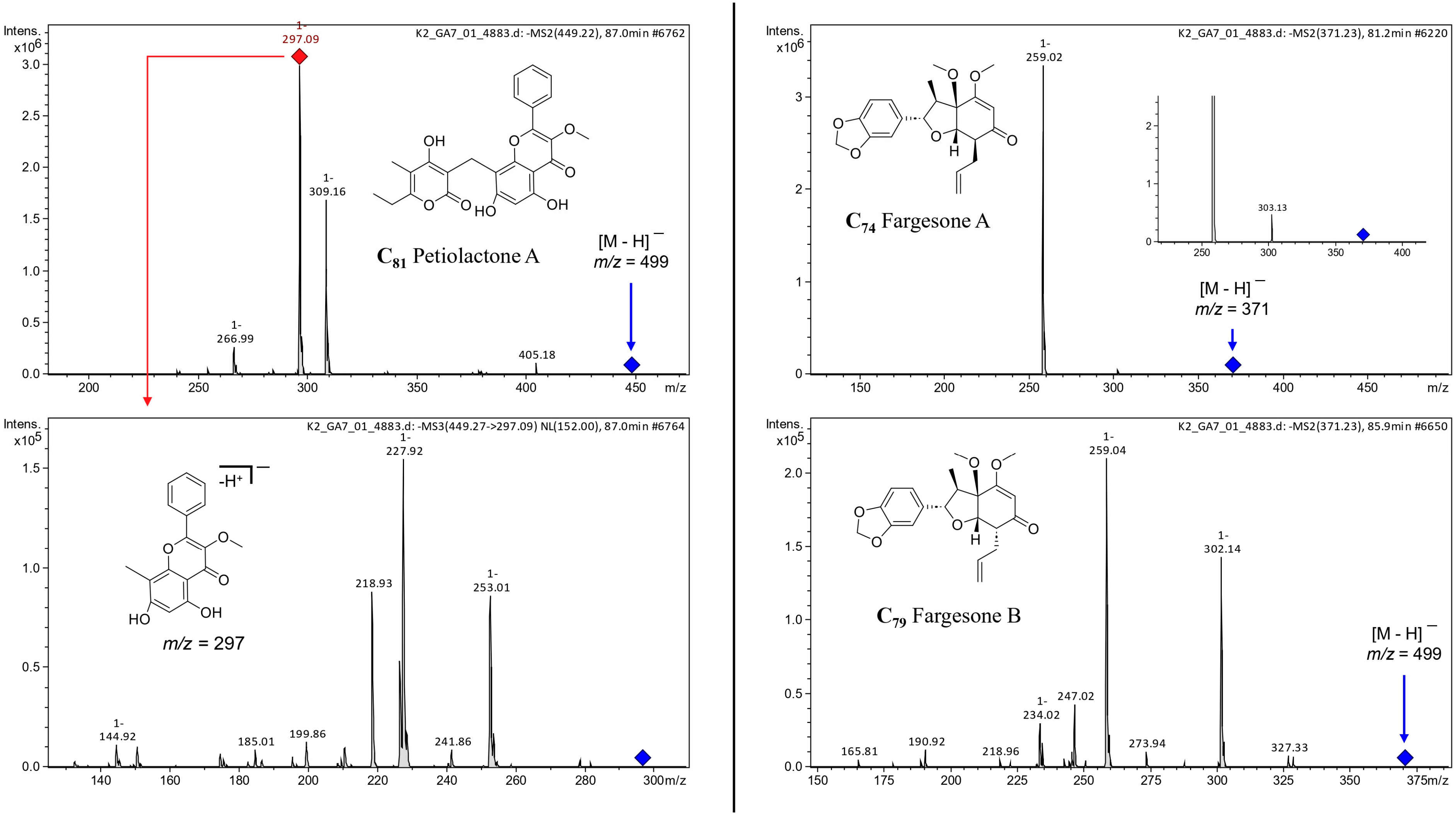

Figure S12. Negative-ion MS² and MS³ spectra of compound C_{81}, tentatively identified as petiolactone A, with the proposed structure of a selected product ion, and negative-ion MS² spectra of compounds C_{74} and C_{79}, tentatively identified as fargesone A and fargesone B, respectively.

Table S1. Linearity, LOD, and LOQ data for the used standards.

Analyte	λ_{det} [nm]	Calibration equation	*n*	Correlation coefficient [R^2]	Calibration range [ng per injection]	LOD [ng per injection]	LOQ [ng per injection]
3-*O*-Caffeoylquinic acid	325	y = 5.5738x - 11.6020	8	0.9908	5-500	19.0	57.1
3,5-Di-*O*-caffeoylquinic acid	325	y = 6.8695x - 86.3454	8	0.9992	5-500	18.9	58.6
Apigenin	337	y = 13.9816x - 2.7829	7	0.9999	5-250	0.5	1.6

λ_{det} - detection wavelength used during quantification; y - peak area; x - the amount of compound injected onto the column; n - number of concentration levels for the calibration curve; LOD - limit of detection; LOQ - limit of quantification; R^2 -correlation coefficient for the linear model
